# Hospital Acquired Infections Among Solid Organ Transplant Recipients Hospitalized in Intensive Care Unit (2018–2024): A Study of the GiViTI Group

**DOI:** 10.1111/tid.70120

**Published:** 2025-10-23

**Authors:** Camilla Genovese, Martina Offer, Marta Colaneri, Francesca Dore, Giorgia Montrucchio, Giovanni Scaglione, Gianpaola Monti, Alessandra Bandera, Bruno Viaggi, Andrea Gori, Emanuele Palomba, Andrea Lombardi, Stefano Finazzi, the Italian Group for Evaluation of Interventions in Intensive Care Medicine, Fulvio Agostini, Antonello di Paolo, Angelo Pan, Carla Maria Zotti, Costanza Vicentini, Gian Maria Rossolini, Daniela Silengo, Emanuele Russo, Lidia Dalfino

**Affiliations:** ^1^ Department of Infectious Diseases Luigi Sacco Hospital Milan Italy; ^2^ Laboratory of Clinical Data Science Department of Medical Epidemiology Mario Negri Institute for Pharmacological Research IRCCS Ranica Italy; ^3^ Department of Biomedical and Clinical Sciences "L. Sacco" University of Milan Milan Italy; ^4^ Centre for Multidisciplinary Research in Health Science (MACH) University of Milan Milan Italy; ^5^ Department of Anesthesia Intensive Care and Emergency ‘Città della Salute e della Scienza’ Hospital Turin Italy; ^6^ Dipartimento Di Anestesia e Rianimazione ASST Grande Ospedale Metropolitano Niguarda Milan Italy; ^7^ Infectious Diseases Unit IRCCS Ca' Granda Ospedale Maggiore Policlinico Foundation Milan Italy; ^8^ Department of Pathophysiology and Transplantation University of Milan Milan Italy; ^9^ Department of Anaesthesiology Neuro‐Intensive Care Unit Careggi University Hospital Florence Italy; ^10^ Department of Anesthesia Inten sive Care and Emergency ‘Città della Salute e della Scienza’ hospital Turin Italy; ^11^ Department of Clinical and Experimental Medicine University of Pisa Pisa Italy; ^12^ Infectious Diseases Unit Istituti Ospitalieri di Cremona Cremona Italy; ^13^ Department of Public Health and Pae diatrics University of Turin Turin Italy; ^14^ Department of Public Health and Paediatrics Uni versity of Turin Turin Italy; ^15^ Department of Experimental and Clinical Medicine Florence Italy; ^16^ Anesthe sia and Intensive Care Ospedale San Giovanni Bosco Turin Italy; ^17^ Anes thesia and Intensive Care Unit AUSL Romagna- Bufalini Hospital Cesena Italy; ^18^ Anesthesia and Intensive Care Unit Department of Precision and Regenerative Medicine and Ionian Area Polyclinic of Bari, University of Bari Bari Italy

**Keywords:** bacterial infections: ICU, ICU‐acquired infections, MDRO, SOT

## Abstract

**Introduction:**

Limited data exist regarding the burden of intensive care unit (ICU)‐acquired infections in the early post‐solid organ transplant (SOT) period, particularly in multidrug resistant organisms‐endemic settings. This study aims at describing the epidemiology, clinical characteristics, and outcomes of patients who developed an ICU‐acquired infection following a SOT procedure in Italy from 2018 to 2024.

**Methods:**

A multicenter, retrospective study was conducted within the Italian PROSAFE project across 31 ICUs from 2018 to 2024. All adult patients admitted to ICU during the same hospitalization as their organ transplant procedure were included. Bloodstream infections, ventilator associated pneumonia, intra‐abdominal infections, and urinary tract infections occurring more than 48 h after ICU admission were retrieved.

**Results:**

Among 2210 SOT recipients, 154 (6.9%) developed 193 ICU‐acquired infections. Ventilator associated pneumonia was the most frequent (74, 38.3%), followed by bloodstream infections (56, 29%). Multidrug resistant organisms were identified in 34/87 (39%) isolates with available antibiogram. ICU‐acquired infections were associated with significantly higher intra‐ICU mortality (35/154, 22.4% vs. 49/2056, 2.4%; *p *< 0.001) and longer ICU stays (24 vs. 4 days; *p* < 0.001). Patients with infections due to multidrug resistant organisms showed higher mortality and length of stay.

**Conclusions:**

ICU‐acquired infections occurred in nearly 7% of SOT recipients admitted to ICU following a SOT procedure, with a significant contribute of multidrug resistant organisms. These infections were associated with striking differences in mortality and length of stay. Finally, this study suggested that patients with MDRO infections showed trends toward higher mortality and length of stay.

## Graphical abstract

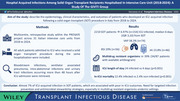

**Abbreviations**
AST: antimicrobial susceptibility testing
BSI: bloodstream infections
CoNS: coagulase‐negative *Staphylococci*
CR‐GNB: carbapenem‐resistant Gram‐negative bacteria
DDI: donor‐derived infection
ECMO: extracorporeal membrane oxygenation
eCRF: electronic case report form
GiViTI: Gruppo Italiano per la Valutazione degli Interventi in Terapia Intensiva
GPB: Gram‐positive bacteria
HAIs: healthcare‐associated infections
IAIs: intra‐abdominal infections
ICU: intensive care unit
LOS: length of stay
MDROs: multidrug‐resistant organisms
PROSAFE: Promoting Patient Safety and Quality Improvement in Critical Care
SOT: solid organ transplant
UTIs: urinary tract infections
VAP: ventilator‐associated pneumonia

## 1 Introduction

The global number of solid organ transplant (SOT) recipients has been steadily increasing, and along with that, both their management and life expectancy have been improved [1]. However, SOT recipients remain at an elevated risk of developing severe infections. This risk is particularly high during the first year after transplantation, especially within the first month post‐surgery. Contributing factors include pre‐existing or latent infections, donor‐derived pathogens, the complexity of the surgical procedure, prosthetic material and/or invasive devices necessary after surgery, as well as prolonged hospital stays often required in the postoperative period [2, 3]. Infections in SOT recipients do not only represent an important cause of death but have been associated with loss of graft or its dysfunction [4].

The prevalence of infections caused by multidrug‐resistant organisms (MDROs) is on the rise, which is associated to higher mortality rates, particularly when carbapenem‐resistant Gram‐negative bacteria (CR‐GNB) are involved [5]. Studies have associated MDRO colonization, both in the donor and in the recipient, to infections [6]. A recent systematic review by Almohaya et al., highlighted the association between CR‐GNB colonization and 1‐year post‐transplant mortality [7]. However, a direct correlation between MDRO colonization and graft loss has yet to be established.

MDRO bloodstream infections (BSIs) are particularly common, with prevalence ranging from 15% to 51% depending on the organ transplanted, and are often accompanied by high mortality [8, 9]. To assess the mortality risk in cases of MDRO‐BSIs, the INCREMENT‐SOT‐CPE score was developed and later validated [10, 11]. Among the included variables, the initiation of an inappropriate empirical therapy plays a significant role in predicting mortality. In fact, patients with MDRO infections frequently receive inadequate empirical antibiotic therapy. Therefore, being aware of local epidemiology, as well as of the risk factors for MDRO colonization and infection is crucial to rapidly initiate the most adequate antimicrobial therapy [12].

SOT recipients admitted to the intensive care unit (ICU) following a SOT procedure, because of their comorbidities, their immunosuppressive status, and the consequences of the surgery they received, face an even greater risk of developing infections caused by MDROs. However, little is known regarding the burden of such infections as well as their outcomes in this population.

With all this in mind, the present study seeks to describe the epidemiology, clinical characteristics, and outcomes of patients admitted to the ICU during the same hospitalization of a SOT procedure who developed healthcare‐associated infections in ICU (ICU‐HAIs) in Italy, a country with high MDRO endemicity, from 2018 to 2024.

## 2 Methods

### 2.1 Study Design

This is a multicenter, retrospective, and observational study conducted within the PROSAFE (Promoting Patient Safety and Quality Improvement in Critical Care) initiative.

The PROSAFE project is a prospective, observational and multicentric study promoted by GiViTI (Gruppo Italiano per la Valutazione degli Interventi in Terapia Intensiva) including more than 200 ICUs [13].

## 3 Objectives

The primary objective of this study was to calculate the incidence of ICU‐HAIs in patients who underwent SOT during hospitalization and who were admitted to the included ICU from 2018 to 2024.

As secondary objectives, the study aimed at evaluating the incidence of MDRO‐ICU‐HAIs over the study period, as well as comparing SOT recipients with MDRO versus non‐MDRO infections in terms of in‐ICU and intra‐hospital length of stay (LOS), mortality, and 28‐day mortality.

### 3.1 Data Collection

Data were consecutively and prospectively collected by ICU physicians, on a voluntary basis, from January 1, 2018, until December 31, 2024, using an electronic case report form (eCRF) provided by GiViTI. Data compilation for each participating center was managed by a designated physician contact person, realistically assisted by approximately two collaborators, for an estimated total of about three ICU physicians per center contributing to data collection in PROSAFE.

To ensure data integrity, cross variable checks were performed during data collection. Validity, according to GiViTI metrics, corresponded to data regarding patients that were admitted in a period (which length depends on the cardinality of the admissions) where at least 90% of patients’ records were complete. ICUs with a reported occupancy rate of less than 50% or with significant heterogeneity in the number of monthly admissions received queries or visits from certified monitors. Following validation, all data from ICUs with at least 4 months of valid data were merged into an aggregated database. Missing data of the interested variables were left as such without applying any imputation methods and were reported in the results.

The collected data encompassed patients’ demographics and comorbidities, as well as data regarding the hospitalization. In addition, information regarding the ICU stay was recorded, including the presence of invasive devices (e.g., endotracheal tube, venous and arterial catheters, urinary catheters), and the need for invasive procedures such as hemodialysis or extracorporeal membrane oxygenation (ECMO, both veno‐venous and veno‐arterial). Data concerning the ICU‐HAI, including the date of onset, the microbiological characteristics, such as the identified pathogens and specific resistance patterns were collected. Finally, in‐ICU and intra‐hospital outcomes, including LOS and mortality rates, were documented.

### 3.2 Inclusion Criteria

All adult SOT recipients admitted to the ICU between January 1, 2018, and December 31, 2024, were included. For the statistical analysis, only patients who remained in the ICU for more than 48 h were considered.

The patient population included in this study reflects the types of transplants performed at the participating centers within the PROSAFE network and is limited to patients admitted to the ICU.

### 3.3 Definitions

SOT recipients were defined as patients who received an organ transplant during the same hospitalization as the ICU admission. For patients who received more than one organ transplant during different hospital admissions, only the first hospital admission was considered. The types of transplants included liver, kidney, lung, heart, and pancreas grafts.

Patients’ comorbidities were classified as follows: respiratory diseases, including chronic obstructive pulmonary disease and restrictive lung diseases; neurological conditions such as cerebral vasculopathy, neuromuscular/neurodegenerative diseases, dementia, encephalopathy, and epilepsy; cardiological diseases, including heart failure, myocardial infarction, hypertension, and peripheral vascular disease; hepatic diseases (e.g., chronic hepatitis and cirrhosis) and chronic kidney disease (CKD), meaning moderate/severe renal insufficiency or end‐stage renal failure.

Regarding the reasons for ICU admission, the “non‐surgical” status refers to the admission in ICU for medical and invasive support only, such as organ failure or acute conditions in patients waiting for SOT or who have received a SOT during the same hospital admission. Instead, “emergency surgical” and “elective surgical” refers to a post‐SOT intensive care management, depending on whether the SOT was performed in emergency or planned.

ICU‐HAIs were defined as infections occurring at least 48 h after ICU admission. For patients who presented an infection prior to, or at ICU admission, only data regarding subsequent infections which developed > 48 h after ICU admission were reported. ICU‐HAIs included BSIs, ventilator‐associated pneumonia (VAP), intra‐abdominal infections (IAIs) and urinary tract infections (UTIs). Specifically, BSIs included all episodes of primary bacteremia (when no recognizable sources are available) and catheter‐related BSIs. IAIs episodes referred to primary/secondary/tertiary peritonitis, post‐surgical peritonitis, cholecystitis/cholangitis, or extra/retroperitoneal abscess. UTIs included non‐surgical UTIs and post‐surgical UTIs.

The diagnosis of ICU‐HAIs was established by specialist intensivist physicians who were in charge of the patients and filled the eCRF, based on two fundamental considerations: 1) the international guidelines provided and recalled by the PROSAFE platform (Supporting Information) [14], and 2) their comprehensive clinical judgment. The infections reported in our study include all clinically diagnosed cases, regardless of whether microbiological isolation was achieved.

Concerning the microbiological data, where available, the most prevalent bacterial pathogens were included, focusing on GNB and Gram‐positive bacteria (GPB). GNB included *Klebsiella* spp., *E. coli*, *Enterobacter* spp., *Proteus* spp., *Citrobacter* spp., *Serratia* spp., *Pseudomonas* spp. and *Acinetobacter* spp., while GPB included *Staphylococcus aureus*, coagulase‐negative *Staphylococci* (CoNS), *Enterococcus* spp. and *Streptococcus pneumoniae*. Resistance, in the case of GNB, was defined as non‐susceptibility to at least one carbapenem (CR). Instead, *Staphylococcus aureus* and CoNS were defined as resistant if non‐susceptible to methicillin, while *Enterococcus* spp. were considered resistant based on vancomycin susceptibility.

### 3.4 Statistical Methods

Categorical data were presented as absolute frequencies and proportions, while continuous variables as mean and standard deviation. Categorical variables were compared with Chi‐square tests, while continuous ones with Mann–Whitney tests. The Cochran–Armitage Trend test was used to assess the presence of trends across ordered categories for binary outcomes. The exact binomial test was employed to compare observed proportions. The statistical significance threshold was set at 0.05. Analyses were performed using R version 4.0.3.

## 4 Results

### 4.1 Patients’ Characteristics

A total of 31 Italian ICUs were involved in this study, of which16 located in hospitals with more, and 9 with less, than 800 beds in 2024 (Table S1). The total number of SOT performed in these centers from 2018 and 2024 was 3880, with some discrepancies between the involved centers (Table S2). The total number of patients was 3720. Among these, 2210 (59.4%) spent at least 48 h in the ICU and were therefore included in the analysis. Patients were aged 58 years (IQR 51, 64) and 1542 (69.8%) were male. Table 1 describes the characteristics of SOT recipients. Through the years, a total of 2304 SOT were performed, the most common transplanted organ was liver (1717, 77.7%), followed by kidney (291, 13.2%), lung (204, 9.2%), and heart (58, 2.6%). The number of procedures increased over the years, except during 2020 and 2021, where it was observed an important reduction of lung and heart transplants due to the Coronavirus disease 2019 pandemic (Table S3). In most cases, SOT occurred the same day as ICU admission (1852, 83.8%). However, in few cases it was performed either before (243, 11%, max 7 days from ICU admission) or during the ICU stay (113, 5.1%, max 47 days from ICU admission).

**TABLE 1:** Demographic and clinic characteristic of SOT‐patients admitted to ICU from 2018 to 2024.

	Overall (*N* = 2210)	Patients with ICU‐HAI (*N* = 154)	Patients without ICU‐HAI (*N* = 2056)	*p* value
**Age (median, IQR)**	58.0 (51.0, 64.0)	57.0 (49.0, 63.8)	58.0 (51.0, 64.0)	0.794
**Male (*n*, %)**	1542 (69.8%)	112 (72.7%)	1430 (69.6%)	0.418
Missing	2	0	2	
**Surgical status**				< 0.001
Non‐surgical	86 (3.9%)	19 (12.3%)	67 (3.3%)	
Elective surgical	197 (8.9%)	9 (5.8%)	188 (9.1%)	
Emergency surgical	1927 (87.2%)	126 (81.8%)	1801 (87.6%)	
**Ward of origin (*n*, %)**				< 0.001
Medical	396 (17.9%)	32 (20.8%)	364 (17.7%)	
Surgical	1645 (74.5%)	97 (63.0%)	1548 (75.4%)	
Emergency	27 (1.2%)	6 (3.9%)	21 (1.0%)	
Other ICU	54 (2.4%)	15 (9.7%)	39 (1.9%)	
High intensity ward	85 (3.9%)	4 (2.6%)	81 (3.9%)	
Missing	3	0	3	
**BMI (median, IQR)**	24.7 (22.3, 27.7)	25.1 (22.5, 28.4)	24.6 (22.3, 27.7)	0.338
Missing	7	0	7	
**Comorbidities (*n*, %)**				
Respiratory disease	452 (20.5%)	38 (24.7%)	414 (20.1%)	0.178
Neurological disease	60 (2.7%)	5 (3.2%)	55 (2.7%)	0.674
Cardiologic disease	1015 (45.9%)	77 (50.0%)	938 (45.6%)	0.293
Chronic kidney disease	373 (16.9%)	26 (16.9%)	347 (16.9%)	0.999
Liver disease	1192 (53.9%)	89 (57.8%)	1103 (53.6%)	0.320
Diabetes	494 (22.4%)	25 (16.2%)	469 (22.8%)	0.059
II‐IV‐degree burn	4 (0.2%)	0 (0.0%)	4 (0.2%)	0.584
Alcohol addiction	132 (6.0%)	7 (4.5%)	125 (6.1%)	0.438
PWID	35 (1.6%)	4 (2.6%)	31 (1.5%)	0.296
**Infections at ICU‐admission (*n*, %)**	15 (0.7%)	15 (9.7%)	0 (0.0%)	< 0.001
**Procedures at admission (*n*, %)**				
CVC	2181 (98.7%)	150 (97.4%)	2031 (98.8%)	0.146
Invasive ventilation	2001 (90.5%)	139 (90.3%)	1862 (90.6%)	0.901
Hemodialysis	41 (1.9%)	5 (3.2%)	36 (1.8%)	0.185
PICC line	21 (1.0%)	1 (0.6%)	20 (1.0%)	0.690
Arterial catheter	2141 (96.9%)	146 (94.8%)	1995 (97.0%)	0.125
Parenteral nutrition	187 (8.5%)	21 (13.6%)	166 (8.1%)	0.017
ECMO	35 (1.6%)	8 (5.2%)	27 (1.3%)	< 0.001
**Intra‐ICU outcomes**				
Mortality (*n*, %)	84 (3.8%)	35 (22.7%)	49 (2.4%)	< 0.001
Length of stay (median, IQR)	4.0 (2.0, 7.0)	24.0 (14.0, 41.5)	4.0 (2.0, 6.0)	< 0.001
**Intra‐hospital outcomes**	Missing = 60	Missing = 11	Missing = 49	
Mortality (*n*, %)	45 (2.1 %)	10 (7.0 %)	35 (1.7 %)	< 0.001
Length of stay (median, IQR)	22.0 (14.0, 40.0)	56.0 (34.5, 85.0)	21.0 (14.0, 36.0)	< 0.001
**Type of transplant** ^a^				
Liver (*n*, %)	1717 (77.7%)	113 (6.6%)	1604 (93.4%)	< 0.001^*^
Lung (*n*, %)	204 (9.2%)	22 (10.8%)	182 (89.2%)	< 0.001^*^
Kidney (*n*, %)	291 (13.2%)	13 (4.5%)	278 (95.5%)	< 0.001^*^
Pancreas (*n*, %)	34 (1.5%)	1 (2.9%)	33 (97.1%)	< 0.001^*^
Heart (*n*, %)	58 (2.6%)	15 (25.9%)	43 (74.1%)	< 0.001^*^

Abbreviations: BMI, body mass index; CVC, central venous catheter; ECMO, extra‐corporeal membrane oxygenation; ICU‐HAI, intensive care unit acquired infection; IQR, interquartile range; PICC, peripherally inserted central catheter; PWID, person who injects drug.

^a^ Patients may have had more than one transplant.

* Exact binomial test (the percentages of the two subgroups ‐infected vs. non‐infected‐ are calculated from the total number of patients for each type of transplant).

### 4.2 Intra‐ICU and Intra‐Hospital Outcomes

An intra‐ICU mortality of 3.8% was observed in this population. SOT recipients who developed an ICU‐HAI experienced an almost 10‐fold higher intra‐ICU mortality with respect to SOT recipients who did not (35, 22.4% vs. 49, 2.4%, *p* < 0.001). Similarly, the intra‐hospital mortality was significantly higher in the ICU‐HAI group (7% vs. 1.7%, *p* < 0.001). Both the intra‐ICU LOS and intra‐hospital LOS were significantly higher in the ICU‐HAI group, with 24 days (IQR 14, 41.5) and 56 days (IQR 34.5, 85), respectively.

Focusing on the MDRO versus non‐MDRO group, although no significant differences were observed in these outcomes, intra‐ICU mortality was higher in the MDRO with respect to the non‐MDRO group reaching 23.5% versus 17%. The intra‐ICU and intra‐hospital LOS were longer in the MDRO group versus the non‐MDRO group (33.5 and 76 days vs. 23 and 52 days, respectively).

### 4.3 Infection Data

A total of 193 infections were acquired by 154 SOT recipients admitted in the ICU, accounting for 6.97% of all SOT patients. Patients developed an ICU‐HAI at a median of 6 days (IQR 4, 14) from ICU admission and of 6 days (IQR 3, 10) from SOT procedure. VAP was the most frequent infection (74, 38.3%), followed by BSI (56, 29%), IAI (46, 23.8%), and UTI (17, 8.8%). Although the prevalence of the different types of infection varied, it did not differ significantly over the years (Table S4).

No significant differences in age, sex and comorbidities were observed between SOT recipients who developed an ICU‐HAI and SOT recipients who did not develop it (non‐ICU‐HAI) (Table 1). However, a greater percentage of patients with an ICU‐HAI were transferred from another ICU (15, 9.7% vs. 39, 1.9%) and were admitted for non‐surgical reasons (19, 12.3% vs. 67, 3.3%). Indeed, 15 (9.7%) SOT recipients who developed an ICU‐HAI were admitted in ICU with an ongoing infection, whereas none of the non‐ICU‐HAI had an ongoing infection at admission. There was no significant difference in invasive devices presence between the ICU‐HAI and non‐ICU‐HAI groups, except for the need of ECMO, which was more frequent in the ICU‐HAI group (8, 5.2% vs. 27, 1.3%, *p* < 0.001). However, only five patients developed eight ICU‐HAIs while on ECMO support. Among these, one VAP and one BSI occurred the same day as the ECMO procedure, while six ICU‐HAIs (four VAP, one BSI, and one UTI) developed while the patients were on ECMO.

**FIGURE 1:**
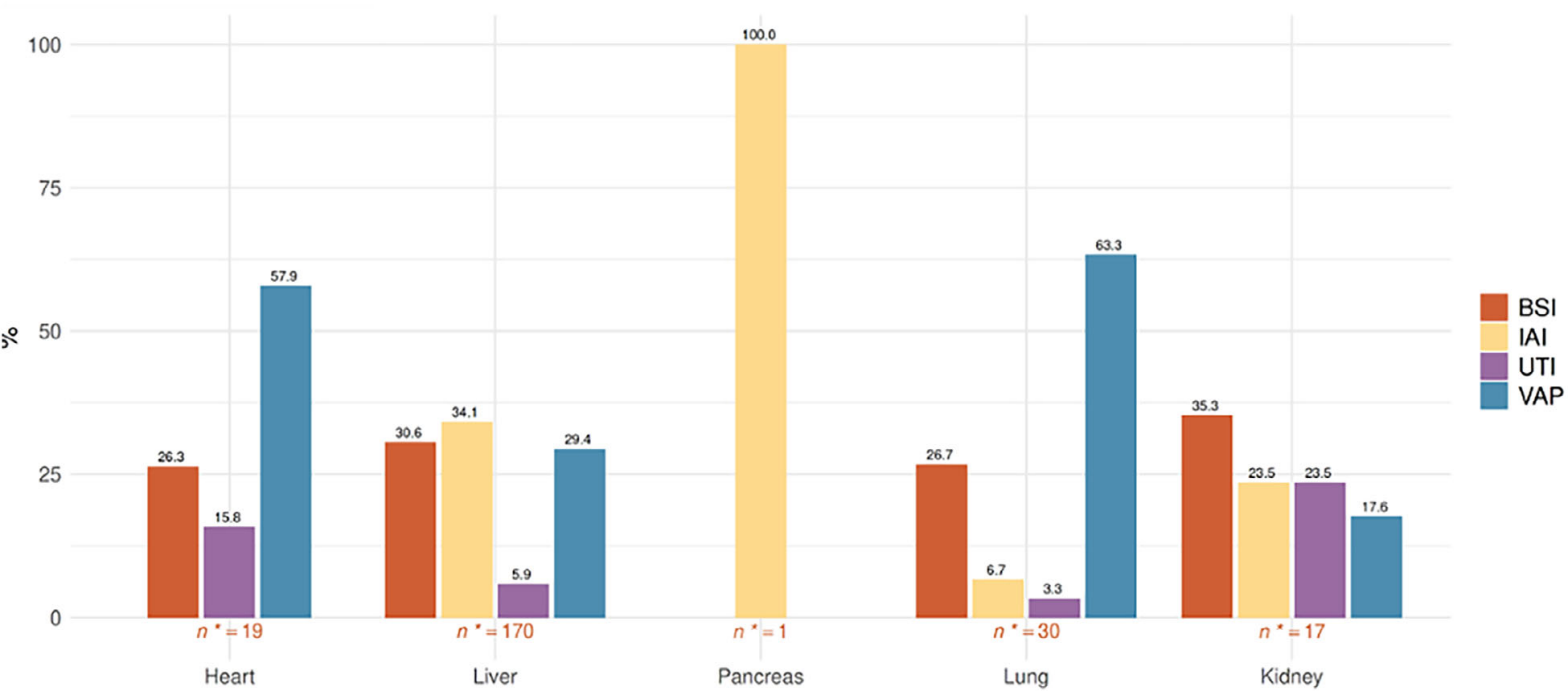
ICU‐HAI for each transplanted organ from 2018 to 2024. BSI, bloodstream infections (catheter‐related bloodstream infections and primary bloodstream infections); IAI, intra‐abdominal infections (primary/secondary/tertiary peritonitis, post‐surgical peritonitis, cholecystitis/cholangitis, extra/retroperitoneal abscess); UTI, urinary tract infections (non‐surgical urinary tract infections, post‐surgical urinary tract infections); VAP, ventilator‐associated pneumonia.

Among the different types of SOT, heart transplant recipients had the greatest incidence of infections (15, 25.9%), followed by lung (22, 10.8%) and liver (113, 6.6%) transplant recipients. VAPs occurred more frequently in heart and lung transplant recipients, representing 57.9% (*n* = 19) and 63.3% (*n* = 19) of ICU‐HAIs for these types of organs, respectively. BSIs occurred with a similar incidence in the different SOTs, ranging from 26.3% (*n* = 5) in heart transplant recipients to 35.3% (*n* = 6) in kidney transplant recipients. IAIs were most often observed in liver, pancreas, and kidney transplant recipients, representing at least a quarter of ICU‐HAIs in these transplants (58, 34.1%, 1, 100%, and 4, 23.5%, respectively). The only ICU‐HAI developed in one pancreas transplant recipient was indeed an IAI. UTI represented a quarter (4, 23.5%) of ICU‐HAIs in kidney transplant recipients, while in the other groups, they were the least frequently developed infections. Figure 1 shows the different frequencies of ICU‐HAIs for each type of SOT.

### 4.4 Microbiological Data

Data regarding antimicrobial susceptibility testing (AST) of the included pathogens were available for 87 (56.5%) patients (Table 2). Of those, 34 (39%) had an MDRO infection, which developed in median 9.5 days (IQR 5.2, 22.8) from the ICU admission. The time to get infected was significantly longer with respect to the non‐MDRO group (6 days, IQR 4, 10). In these subgroups, no significant differences were observed in terms of demographic characteristics and invasive devices' presence. CKD was more frequent in the MDRO‐infected group (10, 29.4% vs. 2, 3.8%, *p* < 0.001), no other significant differences were observed regarding comorbidities. Liver transplant was the largest group in the MDRO‐infected SOT patients (30, 81.1%), followed by kidney (4, 10.8%) and lung transplant (3, 8.1%). In contrast, in the non‐MDRO group, while liver transplant was still the largest (36, 66.6%) group, it was followed by heart (8, 14.8%) and lung (7, 12.9%) transplant recipients. Overall, MDRO‐caused infections were less common compared to non‐MDRO infections (Table S5).

**TABLE 2:** Demographic and clinic characteristic of SOT‐patients with MDRO‐infections versus non‐MDRO infections.

	Patients with at least one MDRO (*N* = 34)	Patients without MDRO (*N* = 53)	*p* value
**Age (median, IQR)**	57.0 (48.0, 63.0)	57.0 (49.0, 63.0)	0.689
**Male (*n*, %)**	23 (67.6%)	43 (81.1%)	0.152
**Admission to ICU**			0.845
Non‐surgical	4 (11.8%)	6 (11.3%)	
Elective surgical	3 (8.8%)	3 (5.7%)	
Emergency surgical	27 (79.4%)	44 (83.0%)	
**Ward of origin (*n*, %)**			0.503
Medical	9 (26.5%)	12 (22.6%)	
Surgical	22 (64.7%)	30 (56.6%)	
Emergency	0 (0.0%)	4 (7.5%)	
Other ICU	2 (5.9%)	4 (7.5%)	
High intensity ward	1 (2.9%)	3 (5.7%)	
**BMI (median, IQR)**	25.5 (21.7, 29.4)	25.1 (22.9, 27.8)	0.858
**Comorbidities (*n*, %)**			
Respiratory disease	7 (20.6%)	10 (18.9%)	0.843
Neurological disease	0 (0.0%)	3 (5.7%)	0.158
Cardiologic disease	15 (44.1%)	29 (54.7%)	0.335
Chronic kidney disease	10 (29.4%)	2 (3.8%)	< 0.001
Liver disease	25 (73.5%)	31 (58.5%)	0.153
Diabetes	8 (23.5%)	8 (15.1%)	0.322
Alcohol addiction	2 (5.9%)	3 (5.7%)	0.956
PWID	1 (2.9%)	2 (3.8%)	0.836
**Infections at ICU‐admission (*n*, %)**	10 (11.5%)	6 (17.6%)	0.150
**Procedures at admission (*n*, %)**			
CVC	34 (100.0%)	51 (96.2%)	0.252
Invasive ventilation	32 (94.1%)	48 (90.6%)	0.552
Hemodialysis	1 (2.9%)	1 (1.9%)	0.749
PICC line	0 (0.0%)	0 (0.0%)	—
Arterial catheter	34 (100.0%)	49 (92.5%)	0.101
Parenteral nutrition	7 (20.6%)	5 (9.4%)	0.141
ECMO	2 (5.9%)	1 (1.9%)	0.391
**Intra‐ICU outcomes**			
Mortality (*n*, %)	8 (23.5%)	9 (17.0%)	0.452
Length of stay (median, IQR)	33.5 (18.0, 54.0)	23.0 (14.0, 37.0)	0.063
Days to get infected (median, IQR)	9.5 (5.2, 22.8)	6.0. (4.0, 10.0)	0.003
**Intra‐hospital outcomes**	Missing = 1	Missing = 6	
Mortality (*n*, %)	2 (6.1%)	3 (6.4%)	0.953
Length of stay (median, IQR)	76.0 (46.0, 94.0)	52.0 (35.5, 76.0)	0.060
**Type of transplant** ^a^			
Liver (*n*, %)	30 (45.5 %)	36 (54.5 %)	0.539^*^
Lung (*n*, %)	3 (30.0 %)	7 (70.0%)	0.344^*^
Kidney (*n*, %)	4 (57.1 %)	3 (42.9 %)	1.000^*^
Pancreas (*n*, %)	0 (0 %)	0 (0 %)	—
Heart (*n*, %)	1 (11.1 %)	8 (88.9 %)	0.039^*^

Abbreviations: BMI, body mass index; CVC, central venous catheter; ECMO, extra‐corporeal membrane oxygenation; ICU, intensive care unit; IQR, interquartile range; MDRO, multidrug‐resistant organism; PICC, peripherally inserted central catheter; PWID, person who injects drug.

^a^ Patients may have had more than one transplant.

* Exact binomial test (the percentages of the two subgroups ‐infected vs. non‐infected‐ are calculated from the total number of patients for each type of transplant).

**FIGURE 2:**
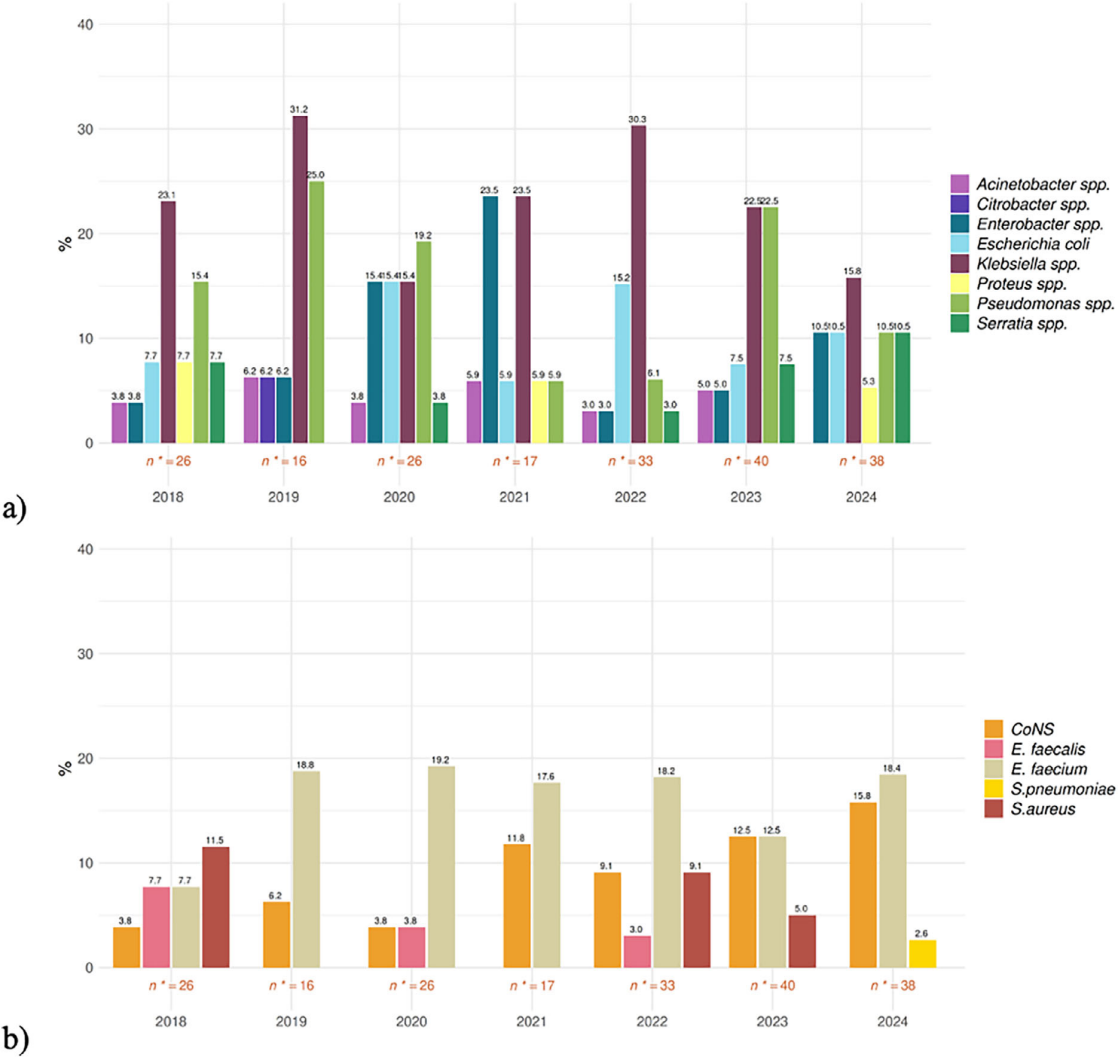
GNB (a) and GPB (b) isolates prevalence in SOT recipients per year. CoNS, coagulase‐negative *staphylococci*; *E. faecalis*, *Enterococcus faecalis*; *E. faecium*, *Enterococcus faecium*; *S. pneumoniae*, *Streptococcus pneumoniae*; *S. aureus*; *Staphylococcus aureus*.

In most cases, ICU‐HAIs were caused by GNB, ranging from 60.6% to 75% through the studied years (Figure S1a). The most common isolated pathogen was *Klebsiella* spp., although, in the latter years a decreasing trend in its prevalence was observed (Figure 2a). The decrease in *Klebsiella* spp. infections was accompanied by an increasing trend in *Enterobacter* spp., *Escherichia coli, Pseudomonas* spp., and *Serratia* spp. infections. The most represented GPB was *Enterococcus faecium*, followed by increasingly prevalent CoNS (Figure 2b). Data regarding antimicrobial susceptibility for each tested pathogen is reported in Figure S1b. The most common MDROs were CR‐*Klebsiella* spp. (12, 34.3% of *Klebsiella* spp. with AST available) and vancomycin resistant *Enterococcus faecium* (14, 56% of *Enterococcus faecium* with AST available). Kidney, lung, and heart transplant recipients only had a small number of reported MDROs (5, 4, and 1, respectively) (Figure S2).

## 5 Discussion

In this large, nationwide study involving more than 30 ICUs, the epidemiology, clinical characteristics and outcomes of SOT recipients who developed an ICU‐HAI during the same hospitalization as their SOT procedure period were described. From 2018 to 2024, a total of 2210 SOT patients were included. Among them, 154 patients experienced 193 ICU‐HAIs, corresponding to an overall incidence of 6.97%.

Although infections occurring within 1‐month post‐transplant are well described in the literature, limited data exist on their ICU‐specific incidence [15]. In this study population, heart transplant recipients showed the highest incidence of ICU‐HAIs, reaching 26%, while lung and liver transplant recipients had lower rates of 10.8% and 6.6%, respectively. This can be explained both by the high degree of immunosuppression to which heart transplant recipients are exposed in the early post‐transplant period and the greater need of invasive devices, such as ECMO [2]. The incidence of ICU‐HAIs in SOT recipients was more than half with respect to the latest European data regarding the general population, which reported a 15% of patients who developed an ICU‐HAI [16]. Similarly, compared with the general ICU population, VAP was the most common ICU‐HAI, followed by BSI [16]. However, IAIs in the SOT population represented the third most common ICU‐HAI with 46 (23.8%) cases, and UTIs were the least common infections. This mirrors the relatively high cumulative incidence of post‐surgical IAIs during ICU stay in the SOT population.

As it might be expected, VAPs were most frequently observed in heart and lung transplant recipients (57.9% and 63.3% of ICU‐HAIs, respectively), while IAIs were more frequent in liver, kidney, and pancreas transplant recipients (34.1%, 23.5%, and 100% of ICU‐HAIs, respectively). UTIs were often seen in kidney transplant recipients representing almost a quarter of ICU‐HAIs in this group (4, 23.5%). These associations may be considered surgical complications, such as anastomotic leaks and the development of IAIs after liver transplant, or VAPs post‐lung transplant, especially in recipients colonized before transplantation [15, 17].

BSIs were observed in approximately one‐third of infected SOT recipients, with an overall incidence of 2.5%, which is lower than the 7.4% reported by a prospective Spanish study [18]. However, that study spanned a longer observation period and was conducted in the early 2000s, when infection prevention practices may have been less advanced. A more recent study reported a similar prevalence of 8.5%, but it included BSIs developed within the first year from SOT [19]. Regarding the infection prevention in SOT hospitalized in the ICU, it must be highlighted that we did not have access to any information about the adopted practices in all the ICUs involved in the study. For instance, the absence of donor clinical and microbiological data precluded the assessment of donor‐derived infection (DDI) risk and the implementation of donor‐guided preventive measures, as recently emphasized by the American Society of Transplantation Infectious Diseases Community of Practice [20]. Although no significant differences were observed in terms of clinical characteristics between SOT recipients who developed an ICU‐HAI and those who did not develop it, ICU‐HAI‐SOT patients were more often transferred from another ICU (9.7% vs. 1.9%) or were initially admitted to ICU for non‐surgical reasons (12.3% vs. 3.3%). In addition, ECMO was required in 5.2% of ICU‐HAI patients compared to 1.3% in the non‐HAI group. This suggests that ICU‐HAI patients may have had more severe conditions at admission or a longer hospitalization history. These patients also had significantly longer ICU LOS of 24 days (IQR 14, 21.5), with respect to 4 days (IQR 2, 6) for non‐ICU‐HAI SOT recipients, as well as a higher intra‐ICU and in‐hospital mortality (22.7% and 7% vs. 2.4% and 1.7%). The nearly 10‐fold increase in in‐ICU mortality and the dramatic difference in ICU LOS underscore the severe clinical impact of nosocomial infections in this vulnerable population. Particularly, given the high incidence of VAP in heart and lung transplant recipients, these findings suggest that intensive, transplant‐specific infection prevention protocols might have substantial impact.

From a microbiological perspective, the most common isolated bacteria were *Klebsiella* spp. and *Pseudomonas* spp. for GNB, while *Enterococcus faecium* and CoNS were the most frequently encountered GPB. This is in line with recent European data regarding ICU‐HAIs, although data regarding specific SOT patients in ICU are lacking [16]. Particularly, in this cohort, 34 (22% of total, 39% of patients with AST) had an MDRO infection. The overall prevalence of CR‐GNB was 25.3% (22/87) in isolates with an available AST. A study conducted in Italy reported a similar prevalence of CR‐GNB infections in SOT patients, with a greater frequency of infections occurring in the early post‐SOT period [21]. Although in the present study it was not possible to establish whether such infections were caused by ICU‐acquired MDROs or whether they developed from a previous known donor or recipient colonization, the literature clearly defines the heightened risk of developing an infection from a known MDRO‐colonization [7]. Therefore, we might suggest a special attention to patients with a known colonization or at risk of developing an MDRO infection [10, 11, 22].

This study has some limitations. First, only SOT patients admitted in ICU during the same hospitalization as their SOT procedure were included, and therefore data regarding ICU‐HAIs in late‐SOT patients could not be evaluated. However, data regarding patients in the first weeks post‐SOT in ICU are poor, and this study represents one of the few available reports. Moreover, the large‐scale, nationwide PROSAFE registry does not include data on the cause of death, and it is not possible to establish reliable proxies for mortality directly attributable to infection. Furthermore, missing AST data in 43.5% of cases represents a substantial limitation. However, this can be attributed to the real‐life, prospective, and voluntary nature of data compilation within the broad PROSAFE registry, which is a general ICU surveillance network not specifically optimized for the SOT population. Missing data may represent either incomplete data field completion by local physicians or genuine lack of microbiological identification (culture‐negative despite clinical signs of infection). Given this limitation, the true prevalence of MDRO infections cannot be definitively established, and our MDRO findings should be interpreted with caution.

Lastly, the study did not capture data on infection prevention measures specific to the recipient population or DDI, which represents an important gap for future research implementations.

## 6 Conclusions

In this large multicentric study, 193 ICU‐HAIs were described in 154 patients who underwent SOT during their hospitalization, resulting in an incidence of 6.97%. In 39% of recipients with available AST, infections were caused by MDROs. ICU‐HAIs infections were associated with striking higher mortality and LOS. Further studies are necessary to evaluate the burden of ICU‐HAIs in this fragile population, particularly including settings with different MDRO epidemiology.

## Author Contributions

**Camilla Genovese**: writing – original draft preparation. **Martina Offer**: methodology, formal analysis and investigation. **Marta Colaneri**: conceptualization. **Francesca Dore**: methodology. **Giorgia Montrucchio**: writing – review and editing. **Giovanni Scaglione**: writing – review and editing. **Gianpaola Monti**: writing ‐ review and editing. **Alessandra Bandera**: supervision. **Bruno Viaggi**: supervision. **Andrea Gori**: supervision. **Emanuele Palomba**: writing – review and editing. **Andrea Lombardi**: conceptualization, writing – original draft preparation. **Stefano Finazzi**: conceptualization, methodology, supervision.

## Ethics Statement

The PROSAFE study protocol was approved by the local ethics committees at the participating centers. The current study has been approved by the scientific committee of GiViTI.

## Conflicts of Interest

Giorgia Montrucchio has received lecture or advisory Board fees from Gilead, Thermofisher, 3M, Grifols; BV has received fees from Abbott, Accelerate Diagnostics, Ada, Advanz Pharma, Alifax, Angelini, Becton Dickinson, Bellco, Biomerieux, Biotest, Cepheid, Correvio, Diasorin, Emmegi Diagnostica, Gilead, InfectoPharm, Menarini, MSD Italia, Nordic Pharma, Pfizer, Shionogi, and Thermofischer Scientific; Alessandra Bandera has received grants for data publication by Gilead, lecture honoraria by Astra Zeneca, Biomérieux, Qiagen, Jassen Cilag, Nordic Pharma, support for attending meetings by Pfizer and Astra Zeneca and advisory board fees by ViiV, Sobi, Gilead and Angelini Pharma; Stefano Finazzi received grants as PI from the Regional Government of Regione Toscana and Regione Piemonte and from MediaClinics Italia S.r.l. for the management of the Electronic Health Record MargheritaTre, European grant United4Surveillance as affiliated entity.

## Supporting information




**Figure S1**: Total number and frequency of Gram‐negative and Gram‐positive isolates per year (a), and antimicrobial resistance prevalence for tested pathogens (b). **Figure S2**: MDR GNB and GPB for each transplanted organ. **Table S1**: Characteristics of the ICUs included in the analysis (2024). **Table S2**: Number of transplants (SOT) stratified by ICUs included in the analysis. **Table S3**: Patients at risk of ICU‐acquired infections by type of transplant per year. **Table S4**: ICU‐acquired infections per year. **Table S5**: Comparison between ICU‐acquired infections with at least one MDRO and without MDRO.

## Data Availability

The complete dataset is available via the corresponding author upon reasonable request.
