# Supplementary material for: Hospital Acquired Infections Among Solid Organ Transplant Recipients Hospitalized in Intensive Care Unit (2018–2024): A Study of the GiViTI Group
Source: Transpl Infect Dis. 2025 Oct 23;27(6):e70120. doi: 10.1111/tid.70120 (PMC12793965; doi:10.1111/tid.70120)
Supplement: Supplementary file 1 — Figure S1: Total number and frequency of Gram‐negative and Gram‐positive isolates per year (a), and antimicrobial resistance prevalence for tested pathogens (b). Figure S2: MDR GNB and GPB for each transplanted organ. Table S1: Characteristics of the ICUs included in the analysis (2024). Table S2: Number of transplants (SOT) stratified by ICUs included in the analysis. Table S3: Patients at risk of ICU‐acquired infections by type of transplant per year. Table S4: ICU‐acquired infections per year. Table S5: Comparison between ICU‐acquired infections with at least one MDRO and without MDRO. [file TID-27-e70120-s001.docx]

***Supplementary material***

**Hospital Acquired Infections Among Solid Organ Transplant Recipients Hospitalized In Intensive Care Unit (2018-2024): A Study Of The GiViTI Group**

Camilla Genovese^1,§^, Martina Offer^1,2,§^, Marta Colaneri^1,3,4*^, Francesca Dore^2^, Giorgia Montrucchio^5^, Giovanni Scaglione^1^, Gianpaola Monti^6^, Alessandra Bandera^7,8^, Bruno Viaggi^9^, Andrea Gori^1,3,4^, Emanuele Palomba^1,4^, Andrea Lombardi^7,8,$^, Stefano Finazzi^2,$^ and the Italian Group for Evaluation of Interventions in Intensive Care Medicine^†,Ф^

^1^Department of Infectious Diseases, Luigi Sacco Hospital, Milan, Italy

^2^Laboratory of Clinical Data Science, Department of Medical Epidemiology, Mario Negri Institute for Pharmacological Research IRCCS, Ranica, Italy

^3^Department of Biomedical and Clinical Sciences "L. Sacco", University of Milan, Milan, Italy
^4^Centre for Multidisciplinary Research in Health Science (MACH), University of Milano, Milano, Italy

^5^Department of Anesthesia, Intensive Care and Emergency ‘Città della Salute e della Scienza’ Hospital, Turin, Italy

^6^Dipartimento di Anestesia e Rianimazione, ASST Grande Ospedale Metropolitano Niguarda, Milan, Italy

^7^Infectious Diseases Unit, IRCCS Ca' Granda Ospedale Maggiore Policlinico Foundation, Milan, Italy

^8^Department of Pathophysiology and Transplantation, University of Milano, Milan, Italy

^9^ Department of Anaesthesiology, Neuro-Intensive Care Unit, Careggi University Hospital, Florence, Italy

^§^These two authors equally contributed.

^$^These two authors equally contributed.

^†^Full list is available in Supplementary Materials.

^Ф^Contributors to tag for PubMed purposes are mentioned in the Acknowledgment section

***Corresponding author**: Marta Colaneri, Department of Infectious Diseases, Luigi Sacco Hospital, Via GB Grassi 74, Milan, Italy, [marta.colaneri@unimi.it](mailto:marta.colaneri@unimi.it) ORCID 0000-0002-5939-9576

**Definitions**

Adapted from: ECDC codebook, Point prevalence survey of healthcare-associated infections and antimicrobial use in European acute care hospitals, European Centre for Disease Prevention and Control, 2022. ECDC Technical document, Surveillance of healthcare-associated infections and prevention indicators in European intensive care units. Stockholm: ECDC; 2017. Sartelli, M., Tascini, C., Coccolini, F. et al. Management of intra-abdominal infections: recommendations by the Italian council for the optimization of antimicrobial use. World J Emerg Surg 19, 23 (2024), <https://doi.org/10.1186/s13017-024-00551-w>. Bassetti M, Eckmann C, Giacobbe DR, Sartelli M, Montravers P. Post-operative abdominal infections: epidemiology, operational definitions, and outcomes. Intensive Care Med. 2020 Feb;46(2):163-172. doi: 10.1007/s00134-019-05841-5.

**Pneumonia**

Radiological criteria:

- Imaging (X-ray/TAC) suggestive of pneumonia

+ Clinical criteria:

At least one of the following:

- Fever >38 °C without other causes or
- Leukopenia (<4000 GB/mm3) or leukocytosis (≥ 12 000 GB/mm3)

At least one of the following:

- New onset purulent sputum or changes in sputum characteristics (colour, odour, quantity, consistency) or
- Cough or dyspnoea or tachypnoea or
- Suggestive lung auscultation (rales or bronchial breath sounds), rhonchi or wheezing or
- Worsening respiratory exchanges (e.g. O_2_ desaturation or increased oxygen requirements or increased ventilation demand)

+ Microbiological criteria:

Microbiological diagnostics performed by:

- Positive quantitative culture from minimally contaminated sample taken from the lower respiratory tract:
  - bronchoalveolar lavage (BAL) with a cut-off value ≥10^4^ colony forming units (CFU)/ml or with >5% cells obtained from BAL containing intracellular bacteria on direct microscopic examination (classified within the BAL diagnostic category)
  - protected brush (PB Wimberley) with a cut-off value ≥10^3^ CFU/ml
  - protected distal aspirate (DPA) with a cut-off value ≥10^3^ CFU/ml
- Positive quantitative culture from possibly contaminated specimen from the lower respiratory tract
  - quantitative culture of lower respiratory tract specimen (e.g. endotracheal aspirate) with a threshold of 10^6^ CFU/ml

Alternatively:

- Positive blood culture not related to another source of infection
- Growth in culture of pleural fluid pleural or pulmonary abscess with positive needle aspiration
- Histologic pulmonary exam shows evidence of pneumonia
- Isolation of specific pathogens (e.g. Legionella, Aspergillus, mycobacteria, mycoplasma, Pneumocystis jirovecii): positive detection of viral antigen or antibody from respiratory secretions (e.g. EIA, FAMA, shell vial assay, PCR), positive direct exam or positive culture from bronchial secretions or tissue, seroconversion (example: influenza viruses, Legionella, Chlamydia), detection of antigens in urine (Legionella)

Other:

- Positive sputum culture or non-quantitative lower respiratory tract specimen culture
- Clinical signs of pneumonia without positive microbiology (microbiology not positive or not performed, or awaiting report)
- Rapid diagnostic tests (e.g. multiplex PCR, syndromic panels)

**Bloodstream infections**

*Primary bloodstream infection of unknown origin*

- Positive blood culture for a recognized pathogen

Alternatively:

- Presence of clinical symptoms (fever >38°C, chills, hypotension)

and

- 2 positive blood cultures for a known skin contaminant pathogen (e.g. coagulase-negative staphylococci, *Micrococcus* spp., *Propionibacterium* *acnes*, *Bacillus* spp., *Corynebacterium* spp.) on two different blood samples.

Of unknown origin means: unknown outbreak of origin or with a different micro-organism with respect to and infection identified elsewhere.

*Catheter related bloodstream infection*

Positive blood culture with the same micro-organism in the following cases:

- Central venous catheter blood culture and catheter tip culture, with quantitative method ≥ 10^3^ CFU/ml or semi-quantitative method >15 CFU
- Delayed differential time of positivity of blood cultures from central venous catheter and peripheral site
- Positive culture from central venous catheter and purulent secretion from insertion site with the same micro-organism

**Urinary tract infections**

*Non-catheter-related urinary tract infection*

Clinical criteria (at least one in the absence of other causes of infection):

- Fever (>38°C)
- Pain/tenderness in suprapubic region
- Pain/tenderness at costovertebral angle
- Pollakiuria
- Dysuria

AND

Laboratory criteria (one of the following):

- Positive urine culture: ≥ 10^5^ CFU/ml urine with no more than two species of microorganisms
- Positive leucocyte esterase and/or nitrites
- Leukocyturia > 10 cells/ml

*Catheter-related urinary tract infection*

Urinary tract infections are considered catheter-related when they occur at least 2 days after bladder catheter placement and within 7 days after its removal.

Clinical criteria (at least one in the absence of other causes of infection):

- Fever (>38°C)
- Pain/tenderness in suprapubic region
- Pain/tenderness at costovertebral angle
- Pollakiuria
- Dysuria

AND

Laboratory criteria (one of the following):

- Positive urine culture: ≥ 10^5^ CFU/ml urine with no more than two species of microorganisms
- Positive leucocyte esterase and/or nitrites
- Leukocyturia > 10 cells/ml

**Intra-abdominal infections**

*Primary peritonitis*

Spontaneous bacterial peritonitis is defined as a bacterial infection of the ascitic fluid without an intra-abdominal source of infection requiring surgical intervention.

*Secondary peritonitis*

Occurs following perforation, abscess formation, ischemic necrosis or penetrating wounds of abdominal organs.

- Leakage of intestinal contents during surgery is not sufficient for diagnosis
- Penetrating abdominal wounds or surgically repaired perforations within 12 hours are not sufficient to diagnose secondary peritonitis

Microbiologically confirmed:

- Isolation of one or more pathogens from the peritoneal fluid or blood within 24 hours of a gastrointestinal perforation (stomach, esophagus, duodenum or small intestine distal to the ligament of Treitz)

Probable:

- Clinical signs with evidence of perforation (free air in the abdomen detected by radiographic examination or surgical confirmation of peritoneal inflammation after perforation)
- Gram-positive staining without positive culture in the peritoneum may be considered as probable secondary peritonitis.

Possible:

- Perforation of the upper gastrointestinal tract or surgically repaired penetrating abdominal trauma, but without microbiological confirmation or other clinical signs of bacterial or fungal peritonitis.
- Presence of peritoneal inflammatory fluid in localized intra-abdominal abscess, but without confirmation by culture.

*Tertiary peritonitis*

Peritonitis that develops following a secondary peritonitis, caused by nosocomial micro-organisms.

Microbiologically confirmed:

- Isolation of nosocomial pathogens from peritoneal fluid or blood, at least 48 hours after treatment of primary or secondary peritonitis

Probable:

- Clinical signs with documented secondary peritonitis and persistent peritoneal inflammation (500 leukocytes/mL peritoneal fluid), but without microbiological confirmation of persistent infection.

Possible:

- Clinical signs with signs of persistent systemic inflammation, but without clear evidence of persistent inflammation in the peritoneal space.

*Post-surgical peritonitis*

Post-operative secondary peritonitis (post-interventional, post-traumatic) is defined as an infectious abdominal complication with peritonitis following a previous surgical intervention that, by definition, requires surgery.

Clinical signs and symptoms:

- Fever
- Abdominal pain and tenderness

Laboratory indicators:

- Increased white blood cell count
- C-reactive protein
- Procalcitonin

Imaging:

- Contrast-enhanced CT scan
- Signs of intestinal perforation, such as extraluminal air and intra-abdominal fluid
- Post-operative abscess

**Table S1.** Characteristics of the ICUs included in the analysis (2024)

|  | **ICUs**  **(N=31)** |
| --- | --- |
| **ICU beds**  Median (Q1, Q3) | 10 (8, 12) |
| *Missing* | *8* |
| **Hospital beds**  Median (Q1, Q3) | 988 (711, 1200) |
| *Missing* | *4* |
| **Hospital type by number of beds**  (n, %) |  |
| Large **(**> 800) | 18 (66.7%) |
| Medium (> 300 & <800) | 9 (33.3%) |
| *Missing* | *4* |
|  |  |

**Table S2.** Number of transplants (SOT) stratified by ICUs included in the analysis.

|  | **SOT**  **(N=3880)** |
| --- | --- |
| **ICU ID** |  |
| 1 | 189 (4.9%) |
| 2 | 2 (0.1%) |
| 3 | 13 (0.3%) |
| 4 | 549 (14.1%) |
| 5 | 3 (0.1%) |
| 6 | 14 (0.4%) |
| 7 | 2 (0.1%) |
| 8 | 6 (0.2%) |
| 9 | 448 (11.5%) |
| 10 | 1 (0.0%) |
| 11 | 4 (0.1%) |
| 12 | 23 (0.6%) |
| 13 | 59 (1.5%) |
| 14 | 48 (1.2%) |
| 15 | 14 (0.4%) |
| 16 | 1 (0.0%) |
| 17 | 1 (0.0%) |
| 18 | 32 (0.8%) |
| 19 | 1 (0.0%) |
| 20 | 62 (1.6%) |
| 21 | 15 (0.4%) |
| 22 | 36 (0.9%) |
| 23 | 30 (0.8%) |
| 24 | 15 (0.4%) |
| 25 | 1 (0.0%) |
| 26 | 152 (3.9%) |
| 27 | 11 (0.3%) |
| 28 | 10 (0.3%) |
| 29 | 11 (0.3%) |
| 30 | 1503 (38.7%) |
| 31 | 624 (16.1%) |

**Table S3.** Patients at risk of ICU-acquired infections by type of transplant per year 

|  | **Total (N=2210)** | **2018 (N=250)** | **2019 (N=271)** | **2020 (N=263)** | **2021 (N=262)** | **2022 (N=331)** | **2023 (N=396)** | **2024 (N=437)** | ***p-value**** |
| --- | --- | --- | --- | --- | --- | --- | --- | --- | --- |
| ***Heart*** | 58 (2.6%) | 13 (5.2%) | 12 (4.4%) | 0 (0.0%) | 0 (0.0%) | 6 (1.8%) | 14 (3.5%) | 13 (3.0%) | *0.391* |
| ***Liver*** | 1717 (77.7%) | 181 (72.4%) | 196 (72.3%) | 242 (92.0%) | 221 (84.4%) | 248 (74.9%) | 291 (73.5%) | 338 (77.3%) | *0.689* |
| ***Pancreas*** | 34 (1.5%) | 0 (0.0%) | 4 (1.5%) | 6 (2.3%) | 6 (2.3%) | 6 (1.8%) | 9 (2.3%) | 3 (0.7%) | *0.641* |
| ***Lung*** | 204 (9.2%) | 24 (9.6%) | 34 (12.5%) | 1 (0.4%) | 20 (7.6%) | 26 (7.9%) | 46 (11.6%) | 53 (12.1%) | *0.047* |
| ***Kidney*** | 291 (13.2%) | 34 (13.6%) | 36 (13.3%) | 33 (12.5%) | 25 (9.5%) | 56 (16.9%) | 61 (15.4%) | 46 (10.5%) | *0.834* |

** Cochran-Armitage trend test*

**Table S4.** ICU-acquired infections per year.

|  | **Total (N=193)** | **2018 (N=26)** | **2019 (N=13)** | **2020 (N=22)** | **2021 (N=20)** | **2022 (N=33)** | **2023 (N=41)** | **2024 (N=38)** | ***p value**** |
| --- | --- | --- | --- | --- | --- | --- | --- | --- | --- |
| ***BSI*** | 56 (29.0%) | 9 (34.6%) | 3 (23.1%) | 7 (31.8%) | 7 (35.0%) | 7 (21.2%) | 14 (34.1%) | 9 (23.7%) | *0.537* |
| ***VAP*** | 74 (38.3%) | 12 (46.2%) | 5 (38.5%) | 7 (31.8%) | 9 (45.0%) | 9 (27.3%) | 16 (39.0%) | 16 (42.1%) | *0.799* |
| ***IAI*** | 46 (23.8%) | 4 (15.4%) | 4 (30.8%) | 7 (31.8%) | 3 (15.0%) | 12 (36.4%) | 6 (14.6%) | 10 (26.3%) | *0.838* |
| ***UTI*** | 17 (8.8%) | 1 (3.8%) | 1 (7.7%) | 1 (4.5%) | 1 (5.0%) | 5 (15.2%) | 5 (12.2%) | 3 (7.9%) | *0.263* |

**Cochran-Armitage trend test*

*BSI, bloodstream infection; VAP, ventilator-associated pneumonia; IAI, intra-abdominal infection; UTI, urinary tract infection.*

**Table S5.** Comparison between ICU-acquired infections with at least one MDRO and without MDRO.

| **ICU-acquired infection with antibiogram** | **At least one MDRO** | **Without MDRO** | **p value*** |
| --- | --- | --- | --- |
| VAP (n = 38) | 9 (23.7 %) | 29 (76.3 %) | 0.002 |
| BSI (n = 43) | 16 (37.2 %) | 27 (62.7 %) | 0.126 |
| IAI (n = 23) | 9 (39.1 %) | 14 (60.9 %) | 0.405 |
| UTI (n = 11) | 4 (36.4 %) | 7 (63.6 %) | 0.549 |

BSI, bloodstream infections (catheter-related bloodstream infections and primary bloodstream infections); IAI, intra-abdominal infections (primary/secondary/tertiary peritonitis, post-surgical peritonitis, cholecystitis/cholangitis, extra/retroperitoneal abscess); UTI, urinary tract infections (non-surgical urinary tract infections, post-surgical urinary tract infections); VAP, ventilator-associated pneumonia 
*Exact binomial test

**
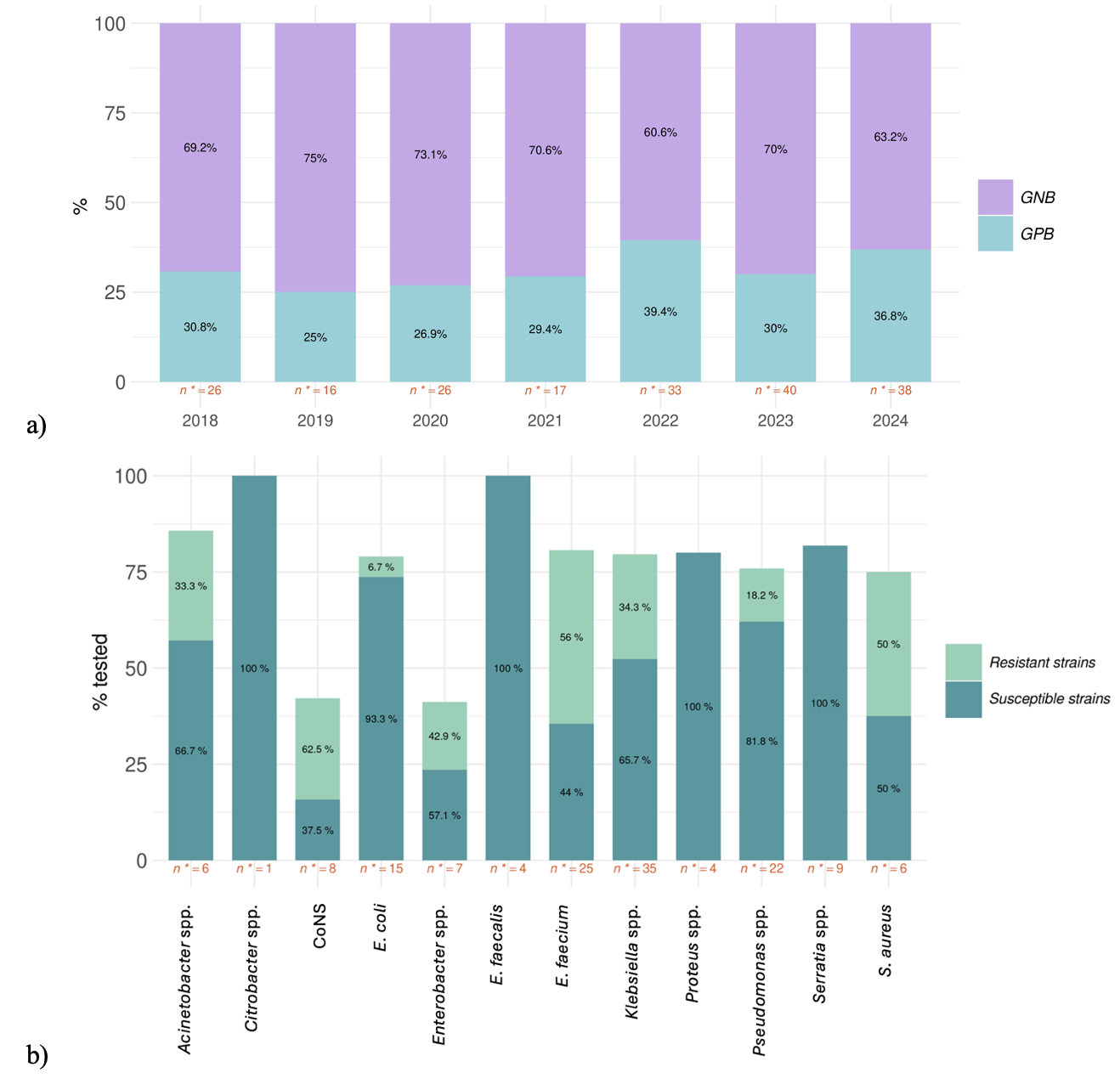
**

**Figure S1.** Total number and frequency of Gram-negative and Gram-positive isolates per year (a), and antimicrobial resistance prevalence for tested pathogens (b).

GNB, Gram negative bacteria included *Klebsiella* spp., *E. coli*, *Enterobacter* spp., *Proteus* spp., *Citrobacter* spp., *Serratia* spp., *Pseudomonas* spp. and *Acinetobacter* spp. GPB, Gram positive bacteria included *Staphylococcus aureus,* coagulase-negative *Staphylococci* (CoNS), *Enterococcus* spp. and *Streptococcus pneumoniae*.

**
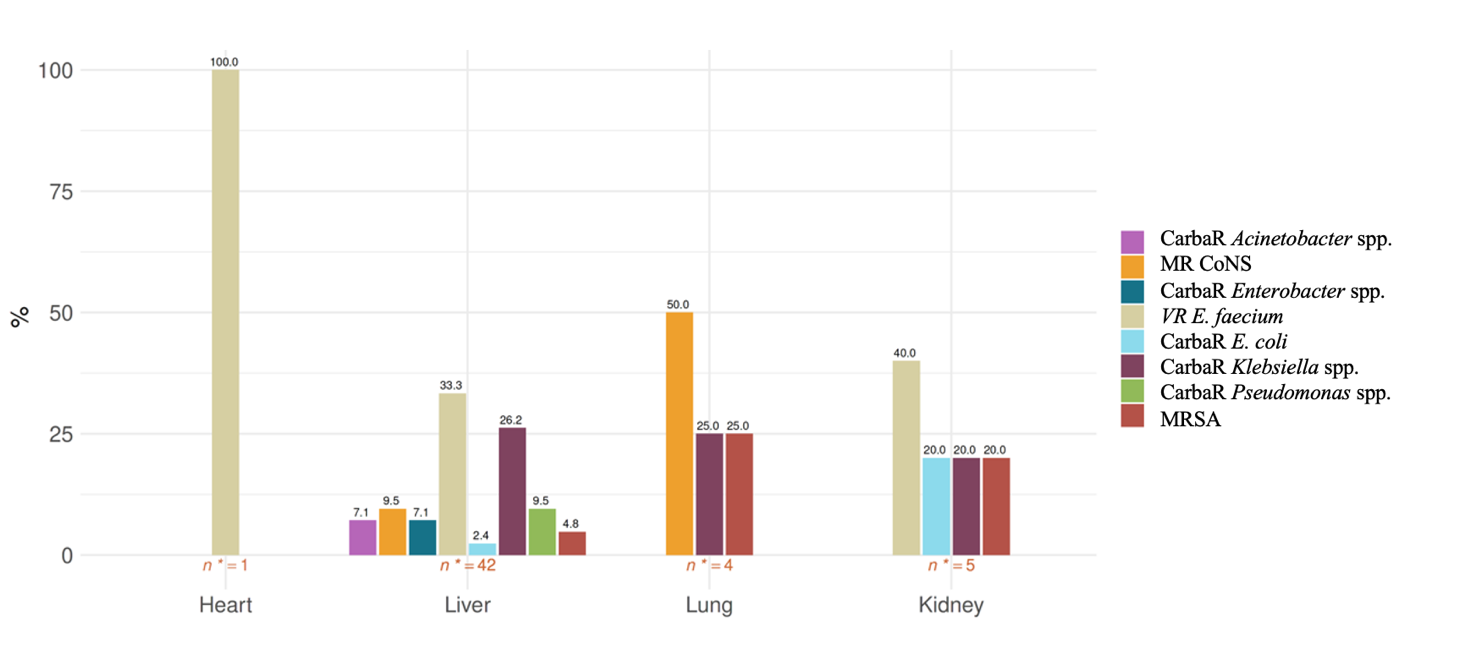
**

**Figure S2.** MDR GNB and GPB for each transplanted organ

*CarbaR, carbapenem resistant; CoNS, coagulase negative* staphylococci; MR; methicillin resistant; *E. faecium, Enterococcus faecium; VR, vancomycin resistant; MRSA, methicillin resistant Staphylococcus aureus*.

**List of Italian Group for Evaluation of Interventions in Intensive Care Unit (GiViTI) contributors**

*Coordinating Center Laboratory of Clinical Data Science, Department of Medical Epidemiology, Mario Negri Institute for Pharmacological Research IRCCS:* Valentina Barbetta, Sara Bettoni, Sara Conti, Francesca Dore, Stefano Finazzi, Elena Garbero, Alice Lavetti, Demetrio Magatti, Martina Offer, Rasoul Samei, Giovanni Tricella.

*Informatics Unit for Sharing Clinical Knowledge, Department of Medical Epidemiology, Mario Negri Institute for Pharmacological Research IRCCS*: Emanuele Colombo, Alessandro Ghilardi, Michele Giardino, Nicolò Mignani, Alberto Savoldelli, Michele Zanetti.

*Scientific and Techincal Committee:* Mario Tavola, GiViTI president; Roberto Fumagalli Ospedale Niguarda, Milano; Università Milano Bicocca, Milano; Carlo Olivieri, Ospedale Sant’Andrea, Vercelli; Anna Zamperoni, Ospedale Cà Foncello, Treviso; Cristiana Cipolla, Ospedale Niguarda, Milano; Bruno Viaggi, Ospedale Careggi, Firenze; Stefano Finazzi, Head of GiViTI Coordinating Center Mario Negri Institute for Pharmacological Research IRCCS; Marco Ranieri, AOU Policlinico di Bari; Adalgisa Caracciolo, Ospedale F. Miulli, Acquaviva delle Fonti (BA); Marco Vergano, Ospedale San Giovanni Bosco, Torino; Arturo Chieregato, Ospedale Niguarda, Milano; Giulia Paci, Alma Mater Studiorum, Università di Bologna,;Aimone Giugni, Ospedale Maggiore, C.A. Pizzardi, Bologna; Lidia Dalfino, AOU Policlinico di Bari; Valentina Barbetta, GiViTI Coordinating Center, Mario Negri Institute for Pharmacological Research IRCCS.

*GiViTI Intensive Care Unit Referents:* Alice Lavetti, GiViTI Coordinating Center, Mario Negri Institute for Pharmacological Research IRCCS; Abascià Arianna, Ospedale Mauriziano Umberto I, Torino (TO); Abastanotti Marco, Ospedale di Manerbio, Manerbio (BS); Acquarolo Annamaria, SPEDALI CIVILI BRESCIA, Brescia (BS); Adorni Adele, Valduce, Como (CO); Agostini Fulvio, AOU Città della Salute e della Scienza di Torino, Torino (TO); Alessandro Gatta, AUSL Romagna - Ospedale di Riccione, Riccione (RN); Alleva Sandra, ASLVCO, P.O. Domodossola, H San Biagio, Domodossola (VB); Alquati Omar, ASST CREMA ex Ospedale Maggiore di Crema, Crema (CR); Amadori Carlo, Azienda Nord Ovest ex 6, Cecina (LI);Amatu Alessandro, Fondazione IRCCS Policlinico S.Matteo, Pavia (PV); Antonini Benvenuto, Ospedale di Manerbio, Manerbio (BS); Archi Davide, Maggiore, Lodi (LO); Arditi Enrico, Ospedale Policlinico San Martino IRCCS PER L'ONCOLOGIA, Genova (GE); Argiolas Federico, Presidio Ospedaliero "Armando Businco", Cagliari (CA); Avalli Leonello, Fondazione IRCCS San Gerardo dei Tintori, Monza (MB); Azan Gaetano, AORN CARDARELLI, Napoli (NA); Azzolini Maurizio, Ospedale Santa Maria del Carmine ROVERETO, Rovereto (TN); Babini Maria, Ospedale Civile Lugo, Lugo (RA); Bagalini Giampiero, Augusto Murri, Fermo (FM); Balestrero Virginia, San Giacomo ASL AL Novi Ligure, Novi Ligure (AL); Balicco Bruno, Policlinico S.Marco Zingonia, Osio Sotto (BG); Baratta Alberto, S Giacomo e Cristoforo, Massa (MS); Barattini Massimo, Azienda USL Toscana Centro - Ospedale Santa Chiara, Firenze (FI); Barbagallo Maria, Azienda Ospedaliero-Universitaria di Parma, Parma (PR); Barboni Elisabetta, Azienda Ospedaliero Universitaria Careggi, Firenze (FI); Bardini Alessandro, ospedale civico carrara, Carrara (MS); Barneschi Chiara, Ospedale del Casentino, Bibbiena (AR); Bartoli Teresa, Ospedale S. M. Annunziata, Bagno A Ripoli (FI); Bassi Francesco, EDOARDO BASSINI, Cinisello Balsamo (MI); Bassi Giovanni, Azienda USL Toscana Nord Ovest - Ospedale delle Apuane, Massa (MS); Battisti Davide, Santa Croce, Fano (PU); Becarelli Simone, usl toscana centro, Prato (PO); Beck Eduardo, ASST Brianza - Ospedale di Desio, Desio (MB); Belgiorno Nicolangela, istituto clinico san rocco di franciacorta, Ome (BS); Bellani Giacomo, APSS - Ospedale S. Chiara, Trento (TN); Bellonzi Alessandra, Azienda Unità Sanitaria Locale di Ferrara, Ferrara (FE); Bendinelli Matteo, USL Toscana Centro, Pistoia (PT); Bensi Marco, Ospedale Civile "SS. Antonio e Margherita", Tortona (AL); Berardino Maurizio, AOU Città della Salute e della Scienza di Torino - Presidio Molinette, Torino (TO); Bernard Moira, San Martino, Belluno (BL); Bernasconi Mara Olga, S. Maria della Misericordia, Rovigo (RO); Berruto Francesco, AOU San Luigi Gonzaga, Orbassano (TO); Berta Giacomo, AOU San Luigi Gonzaga, Orbassano (TO); Bertacchini Sara, Azienda Unità Sanitaria Locale di Ferrara, Ferrara (FE); Bertolini Roberta, Azienda Ospedaliera Universitaria Pisana, Pisa (PI); Bettocchi Daniela, USL Toscana Centro, Prato (PO); Bianchi Tiziana, Fondazione IRCCS Policlinico San Matteo, Pavia (PV); Bignone Paola, S. Croce, Mondovi (CN); Biscione Roberto, Ospedale Nuovo Santa Maria Della Scaletta, Imola (BO);; Boccalatte-Rosa Daniela Luciana, Ospedale Provinciale Di Lucca, Lucca (LU); Bocchi Anna, Policlinico Di Abano Terme, Abano Terme (PD); Bonato Valeria, "Civile - SS Antonio e Biagio e C. Arrigo", Alessandria (AL); Bonazzi Maurizio, I.R.C.C.S. Ospedale Galeazzi Sant'Ambrogio, Milano (MI); Bonicalzi Vincenzo, AOU Città della Salute e della Scienza di Torino - Presidio Molinette, Torino (TO); Bonizzoli Manuela, Azienda Ospedaliero Universitaria Careggi, Firenze (FI); Bonucci Paola, Azienda Ospedaliera Universitaria Senese, Siena (SI); Bosso Riccardo, Azienda Ospedaliero-Universitaria Maggiore della Carità, Novara (NO); Bottazzi Andrea, FONDAZIONE POLICLINICO SAN MATTEO, Pavia (PV); Bottino Nicola, FONDAZIONE IRCCS CA GRANDA OSPEDALE MAGGIORE POLICLINICO, Milano (MI); Brandolini Ilaria, Policlinico Tor Vergata, Roma (RM); Bresadola Francesca, Presidio Ospedaliero Area Nord Bentivoglio-Budrio-San Giovanni Persiceto, Bentivoglio (BO); Breschi Cesare, Azienda Ospedali Riuniti Marche Nord Presidio Di Pesaro, Pesaro (PU); Brizio Elisabetta, OSPEDALE SS ANNUNZIATA, Savigliano (CN); Brunetti Iole, Ospedale Policlinico San Martino IRCCS PER L'ONCOLOGIA, Genova (GE); Brunori Emanuela, OSPEDALE CIVILE MACERATA- AST MACERATA, Macerata (MC); Bruzzone Cristina, ASL 3 Genovese Presidio Ospedaliero VILLA SCASSI, Genova (GE); Buontempo Rosa, San Giovanni di Dio, Orbetello (GR); Buscaglia Giuseppe, IRCCS AZIENDA OSPEDALIERA UNIVERSITARIA SAN MARTINO IST, Genova (GE); Cabrini Luca, Ospedale di Circolo Fondazione Macchi, Varese (VA); Caironi Pietro, AOU San Luigi Gonzaga, Orbassano (TO); Calamai Italo, Ospedale San Giuseppe, Empoli (FI); Calicchio Giuseppe, Azienda Ospedaliera Universitaria San Giovanni di Dio e Ruggi d'Aragona, Salerno (SA); Calò Mauro Antonio, Ospedale Civile Di Mirano, Mirano (VE); Calzolari Alessandro, ASST Ovest Milanese - Ospedale Civile di Legnano, Legnano (MI); Candido Massimo, Azienda Ospedaliero Universitaria Careggi, Firenze (FI); Caporusso Nicola, Francesco Miulli, Bari (BA); Capra Carlo, A.S.S.T. Ovest Milanese - Presidio di Magenta - Ospedale "G. Fornaroli", Magenta (MI); Capuccini Silvia, Spedali Civili di Brescia, Brescia (BS); Caracciolo Adalgisa, Francesco Miulli, Bari (BA); Carli Manuela, USL Toscana Centro, Pistoia (PT); Carnevale Livio, Fondazione IRCCS Policlinico S.Matteo, Pavia (PV); Carrer Sara, ASST-Rhodense - P.O. di Rho, Rho (MI); Casadei Edith, Azienda Ospedaliera Universitaria Senese, Siena (SI); Casadio Maria Cinzia, Ospedale Misericordia Grosseto, Grosseto (GR); Casagli Sergio, Azienda Ospedaliera Universitaria Pisana, Pisa (PI); Casalini Pierpaolo, Per gli Infermi, Faenza (RA); Casalis Michele, USL Toscana Nord Ovest - P.O. Piombino, Piombino (LI); Casella Umberto, AO Ospedale di Circolo di Busto Arsizio Presidio Ospedaliero di Saronno, Saronno (VA); Castelli Gian Paolo, ASST - MANTOVA, Mantova (MN); Castiglione Giacomo, Azienda Ospedalero Universitaria Policlinico "G. Rodolico - San Marco", Catania (CT); Caviglia Enrica, ASL 3 Genovese Presidio Ospedaliero VILLA SCASSI, Genova (GE); Cecchi Alessandra, Ospedale Maggiore, C.A. Pizzardi, Bologna (BO); Centanaro Monica, Ospedale Policlinico San Martino, Genova (GE); Cerana Manuela, Azienda Ospedaliera Ospedale S.Martino e Cliniche Universitarie Convenzionate, Genova (GE); Cerutti Alessandro, "Santa Croce" - Moncalieri - ASL TO 5, Moncalieri (TO); Ciani Andrea, S.S. Cosma e Damiano, Pescia (PT); Ciceri Rita, ASST Lecco, Lecco (LC); Cigada Marco Guido Alberto, ASST Fatebenefratelli Sacco, Milano (MI); Ciocchetti Pierpaolo, Cardinale G. Panìco, Tricase (LE); Clementi Stefano, Ospedale di Sesto San Giovanni, Sesto San Giovanni (MI); Coaloa Maddalena, OSPEDALE SS ANNUNZIATA, Savigliano (CN); Cocciolo Francesco, Maurizio Bufalini, Cesena (FC); Cocco Livio, P.O. "S.Ottone Frangipane", Ariano Irpino (AV); Codazzi Daniela, Fondazione IRCCS Istituto Nazionale dei Tumori, Milano (MI); Colombo Jacopo, ASST Grande Ospedale Metropolitano Niguarda, Milano (MI); Colombo Rinaldo, Ospedale di Circolo Fondazione Macchi, Varese (VA); Colombo Sergio, IRCCS Ospedale San Raffaele, Milano (MI); Corsini Walter, Ospedale Civico Carrara, Carrara (MS); Costagli Valerio, Fondazione IRCCS Istituto Nazionale dei Tumori, Milano (MI); Covani Frigieri Francesca, Ospedale S. M. Annunziata, Bagno A Ripoli (FI); Crema Luciano, Istituti Ospitalieri di Cremona, Cremona (CR); Cristiani Claudia, Azienda Ospedaliera Universitaria Ospedali Riuniti Ancona, Ancona (AN); Curto Francesco Curto, ASST Grande Ospedale Metropolitano Niguarda, Milano (MI); Da Re Dolores, ULSS 6 Euganea, Monselice (PD); Dalfino Lidia, A.U.O POLICLINICO, Bari (BA); De Cristofaro Anna, Azienda Ospedali Riuniti Marche Nord Presidio Di Pesaro, Pesaro (PU); De Gasperi Andrea, ASST Grande Ospedale Metropolitano Niguarda, Milano (MI); De Luca Alessandra, Azienda Ospedaliero Universitaria Careggi, Firenze (FI); De Lucia Marta, Nuovo Ospedale degli Infermi, Ponderano (BI); De Marco Anna Lisa, Nicola Giannettasio, Rossano (CS); De Masi Francesco, Azienda Ospedaliera Universitaria Pisana, Pisa (PI); Dei Poli Marco, Policlinico San Donato, San Donato Milanese (MI); Del Sarto Paolo Antonio Orl, G.Pasquinucci Heart Hospital FTGM, Massa (MS); Della Mora Ernesto, Ospedale Cazzavillan, Arzignano (VI); Della Selva Andrea, Ospedale Michele e Pietro Ferrero-, Verduno (CN); Demozzi Enrico, Ospedale Santa Maria Del Carmine ROVERETO, Rovereto (TN); Di Fini Francesca, Ospedale Buccheri La Ferla, Palermo (PA); Di Masi Pierfrancesco, Istituto di Ricovero e Cura a Carattere Scientifico "Saverio de Bellis", Castellana Grotte (BA); Di Pasquale Dino Aurelio Cleto, Felice Lotti Pontedera, Pontedera (PI); Di Stella Roberta, Ospedale di Circolo Fondazione Macchi, Varese (VA); Donato Stefano, AZIENDA OSPEDALIERA SANTA MARIA, Terni (TR); Doroni Luca, AZIENDA OSPEDALIERA UNIVERSITARIA PISANA, Pisa (PI); Egger Notburga, Santa Croce, Fano (PU); Emmi Vincenzo, FONDAZIONE POLICLINICO SAN MATTEO, Pavia (PV); Erbetta Simona, "Santa Croce" - Moncalieri - ASL TO 5, Moncalieri (TO); Fabbri Emilio, G.B MORGAGNI-L.PIERANTONI, Forlì (FC); Fabbri Lea, Azienda Ospedaliero-Universitaria Careggi, Firenze (FI); Facchini Alberto, ASST Brianza - Ospedale di Desio, Desio (MB); Facondini Francesca, Ospedale "Infermi", Rimini (RN); Faenza Stefano, Policlinico S. Orsola - Malpighi, Bologna (BO); Fagoni Nazzareno, Spedali Civili di Brescia, Brescia (BS); Falini Stefano, Ospedale Misericordia Grosseto, Grosseto (GR); Fanfani Elena, Azienda USL Toscana Centro - Ospedale San Giovanni di Dio - Torregalli, Firenze (FI); Faraldi Loredana, ASST Grande Ospedale Metropolitano Niguarda, Milano (MI); Fernandez Olmos Raquel, Istituto Clinico San Rocco Di Franciacorta, Ome (BS); Ferrari Mariagrazia, Spedali Civili di Brescia, Brescia (BS); Ferretti Marcus, Azienda Ospedaliera Ospedale S.Martino e Cliniche Universitarie Convenzionate, Genova (GE); Ferri Enrico, Ospedale Maggiore, C.A. Pizzardi, Bologna (BO); Ferrucci Giulia, Ospedale Civile SS. Annunziata, Cento (FE); Fiocca Federico, Spedali Civili di Brescia, Brescia (BS); Fiore Gilberto, "Santa Croce" - Moncalieri - ASL TO 5, Moncalieri (TO); Fiorelli Claudio, AZIENDA OSPEDALIERA SANTA MARIA, Terni (TR); Fiume Cosimo, Azienda Ospedaliero-Universitaria Maggiore della Carità, Novara (NO); Fognani Giuliana, Spedali Riuniti Livorno, Livorno (LI); Franchi Federico, Azienda Ospedaliera Universitaria Senese, Siena (SI); Gabini Rita, Ospedale San Donato, Arezzo (AR); Galbiati Rita, Presidio Ospedaliero "Armando Businco", Cagliari (CA); Galeotti Elsa, Ospedale Santa Maria del Prato Feltre, Feltre (BL); Galleschi Nicola, Ospedale San Giuseppe, Empoli (FI); Gallo Mauro, Ospedale Mauriziano Umberto I, Torino (TO); Gamberini Emiliano, AUSL Romagna - Ospedale M.Bufalini, Cesena (FC); Ganzaroli Marco, Ospedale Mauriziano Umberto I, Torino (TO); Garelli Alberto, Santa Maria delle Croci, Ravenna (RA); Garioni Silvia, Azienda ospedaliero-universitaria di Parma, Parma (PR); Gavinelli Veronica, S.S TRINITA', Borgomanero (NO); Genovesi Maurizio, Versilia, Lido Di Camaiore (LU); Gentile Teresa, Azienda Ospedaliera San Camillo Forlanini, Roma (RM); Ghetta Antonella, Azienda Ospedaliera Universitaria Pisana, Pisa (PI); Ghilli Lorenzo, Azienda Ospedaliero Universitaria Careggi, Firenze (FI); Giacomini Matteo, Policlinico S.Marco Zingonia, Osio Sotto (BG); Gianni Massimo, Ospedale Regionale Umberto Parini, Aosta (AO); Giudici Riccardo, ASST Grande Ospedale Metropolitano Niguarda, Milano (MI); Giugni Aimone, Ospedale Maggiore, C.A. Pizzardi, Bologna (BO); Giuntini Romano, Ospedale San Giuseppe, Empoli (FI); Giuntoli Monica, Spedali Riuniti Livorno, Livorno (LI); Gorietti Adonella, Santa Maria della Misericordia, Perugia (PG); Greco Maurizio, P.O. "S.Ottone Frangipane", Ariano Irpino (AV); Greco Stefano, AO Ospedale di Circolo di Busto Arsizio Presidio Ospedaliero di Saronno, Saronno (VA); Grioni Andreina, Ospedale A.Uboldo Presidio Ospedaliero Cernusco s/N, Cernusco Sul Naviglio (MI); Guadagnucci Alberto, G.Pasquinucci Heart Hospital FTGM, Massa (MS); Guagliardi Clementina, S. Antonio Abate, Gallarate (VA); Guarducci Maria Diletta, Ospedale S. M. Annunziata, Bagno A Ripoli (FI); Guarracino Fabio, AZIENDA OSPEDALIERA UNIVERSITARIA PISANA, Pisa (PI); Guerra Emmanuele, Nuovo Ospedale Civile S.Agostino Estense, Modena (MO); Guffanti Elena Angela Augusta, ASST Grande Ospedale Metropolitano Niguarda, Milano (MI); Guido Stefania, AOU "Maggiore della Carità", Novara (NO); Isetta Michele, ASL 3 Genovese Presidio Ospedaliero VILLA SCASSI, Genova (GE); Jorio Antonella, Area Vasta 2, Jesi (AN); Kola Dea, Policlinico Di Abano Terme, Abano Terme (PD); Lacquaniti Luigi, Nicola Giannettasio, Rossano (CS); Laici Cristiana, Policlinico S. Orsola - Malpighi, Bologna (BO); Lamborghini Sara, Azienda Ospedaliero-Universitaria di Ferrara, Arcispedale S. Anna, Ferrara (FE); Langer Martin, Fondazione IRCCS Istituto Nazionale dei Tumori, Milano (MI); Lanza Giuseppina, Ospedale Michele e Pietro Ferrero-, Verduno (CN); Lanza Maria Concetta, G.B MORGAGNI-L.PIERANTONI, Forlì (FC); Lefons Ugo, Ospedale Alta Val d'Elsa, Poggibonsi (SI); Leggieri Carlo, IRCCS Ospedale San Raffaele, Milano (MI); Legnani Martino Gregorio, Ospedale Civile SS. Annunziata, Cento (FE); Lembo Rita, Ospedale Castelli di Verbania, Verbania (VB); Librenti Marco, Azienda USL Toscana Centro - Ospedale San Giovanni di Dio - Torregalli, Firenze (FI); Ligi Silvia, Azienda ospedali Riuniti marche Nord presidio di Pesaro, Pesaro (PU); Liverani Chiara Maria, Ospedale di Sesto San Giovanni, Sesto San Giovanni (MI); Lomagistro Marina, IRCSS Casa Sollievo della Sofferenza, San Giovanni Rotondo (FG); Loriga Beatrice, Ospedali Riuniti Valdichiana Senese Sud, Montepulciano (SI); Madeira Susana Monica, Ospedale del Casentino, Bibbiena (AR); Madonna Roberto, Ospedale Misericordia Grosseto, Grosseto (GR); Maestrone Carlo, ASLVCO, P.O. Domodossola, H San Biagio, Domodossola (VB); Magatti Mariafederica, Azienda Ospedaliera S.ANNA- Ospedale S.ANNA, San Fermo Della Battaglia (CO); Malacarne Paolo, Azienda Ospedaliera Universitaria Pisana, Pisa (PI); Mamprin Filippo, A.O. Bolognini - Seriate, Seriate (BG); Mangani Valerio, Azienda USL Toscana Centro - Ospedale San Giovanni di Dio - Torregalli, Firenze (FI); Mannolini Giovanni, Ospedale "S.Antonio Abate", Pontremoli (MS); Mantovani Giorgio, Azienda Ospedaliero-Universitaria di Ferrara, Arcispedale S. Anna, Ferrara (FE); Manzi Renato Carlo, Fondazione IRCCS Istituto Nazionale dei Tumori, Milano (MI); Marafon Silvio, Ospedale Cazzavillan, Arzignano (VI); Marcello Maria Emilia, Azienda Ospedaliera Brotzu, Cagliari (CA); Mariconti Laura, Maggiore, Lodi (LO): Marini Federica, Ospedale Alta Val d'Elsa, Poggibonsi (SI); Marino Giovanni, Azienda Ospedaliera di Melegnano Presidio di Vizzolo Predabissi, Vizzolo Predabissi (MI); Martin Marina Alessandra, San Bortolo, Vicenza (VI); Martinelli Paola, Ospedale Castelli di Verbania, Verbania (VB); Martinelli Paolo, Azienda Ospedaliero-Universitaria Careggi, Firenze (FI); Martinez Escobar Ricardo, Fondazione Poliambulanza Istituto Ospedaliero, Brescia (BS); Marudi Andrea, Nuovo Ospedale Civile Sant'Agostino Estense, Modena (MO); Marzullo Antonella, AOU Città della Salute e della Scienza di Torino - Presidio Molinette, Torino (TO); Masia Caterina, Nuovo Ospedale degli Infermi, Ponderano (BI); Mastroianni Alessandro, MAGGIORE, Chieri (TO); Maurelli Marco, Fondazione IRCCS Policlinico San Matteo, Pavia (PV); Mazzoccone Antonio, Azienda Ospedaliero-Universitaria Careggi, Firenze (FI); Mazzoleni Patrizia, Azienda Ospedaliera Brotzu, Cagliari (CA); Mediani Teresa Sabina, FONDAZIONE POLICLINICO SAN MATTEO, Pavia (PV); Melani Dario, USL9, Grosseto (GR); Melis Martina, Giovanni Paolo II, Olbia (OT); Mengoli Francesca, Ospedale Maggiore, C.A. Pizzardi, Bologna (BO); Mereto Nadia, ASL 3 Genovese - P.O. Villa Scassi, Genova (GE); Messina Marianna, Giovanni Paolo II, Olbia (OT); Micheli Fabio, A.O.Papa Giovanni XXIII, Bergamo (BG); Militano Carmine Rocco, Fondazione Poliambulanza - Istituto Ospedaliero, Brescia (BS); Minnucci Francesco, OSPEDALE CIVILE MACERATA- AST MACERATA, Macerata (MC); Molesi Andrea, Area Vasta 2, Jesi (AN); Monfregola Maria Rosaria, Azienda Ospedaliera Universitaria Senese, Siena (SI); Monge Roffarello Claudia Angela, OSPEDALE SS ANNUNZIATA, Savigliano (CN); Mongelli Pierpaolo, Azienda Ospedaliera Universitaria Senese, Siena (SI); Montillo Gerardo, Nicola Giannettasio, Rossano (CS); Monzani Norma, EDOARDO BASSINI, Cinisello Balsamo (MI); Morelli Sandro Morelli, AZIENDA OSPEDALIERA SANTA MARIA, Terni (TR); Morosini Paolo, Area Vasta 2 ASUR MARCHE, Fabriano (AN); Mucci Milena, IRCCS Ospedale San Raffaele, Milano (MI); Munafò Anna Maria, Azienda Ospedalero Universitaria Policlinico "G. Rodolico - San Marco", Catania (CT); Munari Marina, A.O. di Padova, Padova (PD); Munaron Susanna, OSPEDALE SAN GIACOMO, Castelfranco Veneto (TV); Musso Stefania Musso, ASO Santa croce e Carle, Cuneo (CN); Napoleone Alessandra, Azienda Ospedaliera Brotzu, Cagliari (CA); Nardi Giuseppe, azienda ospedaliera san camillo forlanini, Roma (RM); Nardin Giordano, ASL Taranto, Taranto (TA); Nardini Massimiliano, Versilia, Lido Di Camaiore (LU); Nascimben Ennio, Ospedale Ca' Foncello di Treviso, Treviso (TV); Natalini Giuseppe, Fondazione Poliambulanza - Istituto Ospedaliero, Brescia (BS); Nava Luana, ASL 3 Genovese - P.O. Villa Scassi, Genova (GE); Negri Giovanni, A.S.S.T. Ovest Milanese - Presidio di Magenta - Ospedale "G. Fornaroli", Magenta (MI); Negro Giancarlo, ASLE LECCE - PRESIDIO OSPEDALIERO GALLIPOLI, Gallipoli (LE); Neri Massimo, IRCCS ISTITUTO DI SCIENZE NEUROLOGICHE - OSPEDALE BELLARIA, Bologna (BO); Nucci Maria Letizia, Azienda Ospedaliera Universitaria Senese, Siena (SI); Odetto Lorenzo, AOU San Luigi Gonzaga, Orbassano (TO); Olivieri Carlo, AOU "Maggiore della Carità", Novara (NO); Olivieri Maria Candida, ospedale san donato, Arezzo (AR); Padovan Lamberto, ULSS 6 euganea, Monselice (PD); Paganoni Guido, A.O.Papa Giovanni XXIII, Bergamo (BG); Paracchini Simone, Ospedale Provinciale Di Lucca, Lucca (LU); Parnigotto Alessandra, ULSS 6 euganea, Monselice (PD); Parrini Vieri, OSPEDALE DEL MUGELLO, Borgo San Lorenzo (FI); Pasculli Marcello, Azienda Ospedaliera Universitaria Senese, Siena (SI); Pasetti Giovanni Stefano, San Giovanni di Dio, Orbetello (GR); Pastorini Simonetta, Ospedale P. Cosma-AUSL 15 Alta padovana, Camposampiero (PD); Pavoni Vittorio, Ospedale S. M. Annunziata, Bagno A Ripoli (FI); Pedeferri Matteo, Azienda Ospedaliera della Provincia di Lecco - Presidio Ospedaliero "S. Leopoldo Mandic" Merate, Merate (LC): Pegoraro Maurizio, OSPEDALE SAN GIACOMO, Castelfranco Veneto (TV); Pera Laura, Azienda USL Toscana Centro - Ospedale Santa Chiara, Firenze (FI); Pero Alice, S.ANDREA, Vercelli (VC); Pessano Paolo, APSS - Ospedale S. Chiara, Trento (TN); Peta Mario, Ospedale Ca' Foncello di Treviso, Treviso (TV); Petrucci Nicola, Azienda Socio-Sanitaria Territoriale del Garda, Presidio di Desenzano, Desenzano Del Garda (BS); Pettazzi Giorgio, Ospedale Civile "SS. Antonio e Margherita", Tortona (AL); Pezzi Angelo, EDOARDO BASSINI, Cinisello Balsamo (MI); Pezzi Giuseppe, Per gli Infermi, Faenza (RA); Piccinini Paolo, Nuovo Ospedale Civile Sant'Agostino Estense, Modena (MO); Piccioni Giuseppe, Spedali Civili di Brescia, Brescia (BS); Pierelli Daniele, Azienda Ospedaliero-Universitaria Maggiore della Carità, Novara (NO); Pifferi Silvia, Fondazione IRCCS Ca' Granda Ospedale Maggiore Policlinico, Milano (MI); Pignatti Alessandro, USL Modena - Ospedle Civile 'B.Ramazzini', Carpi (MO); Pinna Cristina, Nuovo Ospedale Civile S.Agostino Estense, Modena (MO); Pintucci Rita, ASST Fatebenefratelli Sacco, Milano (MI); Pisu Marina, Azienda Ospedaliera Brotzu, Cagliari (CA); Piva Simone, SPEDALI CIVILI BRESCIA, Brescia (BS); Pizzaballa Maria Luigia, Policlinico S.Marco Zingonia, Osio Sotto (BG); Pizzali Mario, Ospedale Civile Di Mirano, Mirano (VE); Platini Mariagrazia, Ospedale Castelli di Verbania, Verbania (VB); Polieri Debora, Di Venere, Bari (BA); Pompili Antonella, AUSL Romagna - Ospedale di Riccione, Riccione (RN); Poole Daniele, San Martino, Belluno (BL); Porcile Elisa, Ospedale Policlinico San Martino, Genova (GE); Prato Maurizio, San Giacomo ASL AL Novi Ligure, Novi Ligure (AL); Pressato Lorenzo Simone, ASST Grande Ospedale Metropolitano Niguarda, Milano (MI); Prosperi Manlio, ASST Grande Ospedale Metropolitano Niguarda, Milano (MI); Psimadas Ioannis, Ospedale Civile Di Mirano, Mirano (VE); Pulici Marco, ASST Grande Ospedale Metropolitano Niguarda, Milano (MI); Raffa Stefania, ASLVCO, P.O. Domodossola, H San Biagio, Domodossola (VB); Rambaldi Marco, Nuovo Ospedale Civile S.Agostino Estense, Modena (MO); Randellini Roberto, Ospedali Riuniti Valdichiana Senese Sud, Montepulciano (SI); Ranzini Luisa, ASST Grande Ospedale Metropolitano Niguarda, Milano (MI); Recchia Andreaserena, IRCCS Casa Sollievo della Sofferenza, San Giovanni Rotondo (FG); Righini Erminio, Ospedale del Delta - Azienda USL Ferrara, Lagosanto (FE); Riva Alberto, Azienda Ospedaliera della Provincia di Lecco - Presidio Ospedaliero "S. Leopoldo Mandic" Merate, Merate (LC); Rizzi Maurizio, A.O. Bolognini - Seriate, Seriate (BG); Rizzi Tatiana, ASST-Rhodense - P.O. di Rho, Rho (MI); Robbiati Alessandro, ASST-Rhodense - P.O. di Rho, Rho (MI); Roberto Eugenia, Felice Lotti Pontedera, Pontedera (PI); Romito Francesco Massimo, Madonna delle Grazie, Matera (MT); Rona Roberto, Fondazione IRCCS San Gerardo dei Tintori, Monza (MB); Rosano Antonio, Fondazione Poliambulanza - Istituto Ospedaliero, Brescia (BS); Rosanò Elisabetta, Azienda Ospedaliera Universitaria Ospedali Riuniti Ancona, Ancona (AN); Rosi Roberto, Azienda Ospedaliera Universitaria Senese, Siena (SI); Rossi Maurizio, ASST Lariana, Menaggio (CO); Rossi Simona, ASST-Rhodense - P.O. di Rho, Rho (MI); Roticiani Valeria, Ospedale Santa Maria alla Gruccia, Montevarchi (AR); Ruggeri Patrizia, Istituti Ospitalieri di Cremona, Cremona (CR); Russo Emanuele, AUSL Romagna - Ospedale M.Bufalini, Cesena (FC); Sabbatini Giovanni, EDOARDO BASSINI, Cinisello Balsamo (MI); Saccavino Erica, ASST Brianza - Ospedale di Desio, Desio (MB); Sacchi Marco, ASST Grande Ospedale Metropolitano Niguarda, Milano (MI); Sagliaschi Ugo, S.S TRINITA', Borgomanero (NO); Salvi Giovanni, Presidio Ospedaliero di Imperia, Imperia (IM); Sambuco Monica, Ospedale di Circolo Fondazione Macchi, Varese (VA); Santambrogio Luisa, Ospedale Castelli di Verbania, Verbania (VB); Sara Calzolari, OSPEDALE DEL MUGELLO, Borgo San Lorenzo (FI); Scarrone Silvia, "Civile - SS Antonio e Biagio e C. Arrigo", Alessandria (AL); Schillaci Maria, Ospedale A.Uboldo Presidio Ospedaliero Cernusco s/N, Cernusco Sul Naviglio (MI); Scibilia Giuseppina, I.R.C.C.S. Ospedale Galeazzi Sant'Ambrogio, Milano (MI); Segala Vincenzo, Ospedale Mauriziano Umberto I, Torino (TO); Seno Alberto, APSS - Ospedale S. Chiara, Trento (TN); Serio Daniela, ASL Taranto, Taranto (TA); Serra Luciano, Presidio Ospedaliero "Armando Businco", Cagliari (CA); Sicignano Alberto, FONDAZIONE IRCCS CA GRANDA OSPEDALE MAGGIORE POLICLINICO, Milano (MI); Soldà Paola Rosa, ASLVCO, P.O. Domodossola, H San Biagio, Domodossola (VB); Sorbara Carlo, Ospedale Ca' Foncello di Treviso, Treviso (TV); Sorgato Cristina, policlinico di abano terme, Abano Terme (PD), Spadini Elisabetta, Azienda Ospedaliero-Universitaria di Parma, Parma (PR); Spagarino Ermanno, Nuovo Ospedale degli Infermi, Ponderano (BI); Speciale Roberto, ASST Lecco, Lecco (LC); Storti Enrico, Maggiore, Lodi (LO); Sucre Maria José, San Leonardo, Castellammare Di Stabia (NA); Tavola Mario, ASL 3 Genovese Presidio Ospedaliero VILLA SCASSI, Genova (GE); Tenio Rita, S. Croce, Mondovi (CN); Terzitta Marina, G.B MORGAGNI-L.PIERANTONI, Forlì (FC); Testa Marco, OSPEDALE SS ANNUNZIATA, Savigliano (CN); Tini Laura, Ospedale dell'Angelo, Venezia (VE); Tintori Davide, Spedali Civili di Brescia, Brescia (BS); Tinturini Rebecca, Azienda Ospedaliera Universitaria Senese, Siena (SI); Tofani Rossella, Spedali Riuniti Livorno, Livorno (LI); Tomaselli Paola, Fondazione IRCCS Ca' Granda Ospedale Maggiore Policlinico, Milano (MI); Toscani Monica, Fondazione IRCCS Policlinico San Matteo, Pavia (PV); Tosi Luigi, Ospedale "S.Antonio Abate", Pontremoli (MS); Turchet Federica, OSPEDALE SAN GIACOMO, Castelfranco Veneto (TV); Turriziani Ilaria, Ospedale Maggiore, C.A. Pizzardi, Bologna (BO); Urbino Rosario, AOU Città della Salute e della Scienza di Torino - Presidio Molinette, Torino (TO); Vaccari Caterina, San Giacomo ASL AL Novi Ligure, Novi Ligure (AL); Vaccarini Barbara, San Luca, Trecenta (RO); Vaj Monica, AOU Città della Salute e della Scienza di Torino - Presidio Molinette, Torino (TO); Vardanega Andrea, Ospedale dell'Angelo, Venezia (VE); Varelli Giancarlo, Azienda Ospedaliera Universitaria Pisana, Pisa (PI); Vason Milo, Azienda Ospedaliero-Universitaria di Ferrara, Arcispedale S. Anna, Ferrara (FE); Vecchiarelli Ada, Santa Maria della Misericordia, Perugia (PG); Ventura Luciana, "Civile - SS Antonio e Biagio e C. Arrigo", Alessandria (AL); Venturini Elisabetta, "Civile - SS Antonio e Biagio e C. Arrigo", Alessandria (AL); Vespignani Maria Giovanna, ospedale nuovo santa maria della scaletta, Imola (BO); Vincenzi Matteo, IRCCS ISTITUTO DI SCIENZE NEUROLOGICHE - OSPEDALE BELLARIA, Bologna (BO); Visconti Maria Grazia, Ospedale A.Uboldo Presidio Ospedaliero Cernusco s/N, Cernusco Sul Naviglio (MI); Voto Giuliana, San Leonardo, Castellammare Di Stabia (NA); Vulcano Giuseppe Angelo, Nicola Giannettasio, Rossano (CS); Zani Gianluca, Santa Maria delle Croci, Ravenna (RA); Zanni Vittorio, presidio ospedaliero area nord bentivoglio-budrio-san giovanni persiceto, Bentivoglio (BO); Zardin Michela, APSS - Ospedale S. Chiara, Trento (TN); Zompanti Valeria, OSPEDALE CIVILE MACERATA- AST MACERATA, Macerata (MC); Zonta Giandomenico, Azienda Socio-Sanitaria Territoriale del Garda, Presidio di Desenzano, Desenzano Del Garda (BS); Zoppellari Roberto, Azienda Ospedaliero-Universitaria di Ferrara, Arcispedale S. Anna, Ferrara (FE); Zuccaro Francesco, Madonna delle Grazie, Matera (MT).
